# *Moving through the Stressed Genome*: Emerging Regulatory Roles for Transposons in Plant Stress Response

**DOI:** 10.3389/fpls.2016.01448

**Published:** 2016-10-10

**Authors:** Pooja Negi, Archana N. Rai, Penna Suprasanna

**Affiliations:** Plant Stress Physiology and Biotechnology Section, Nuclear Agriculture and Biotechnology Division, Bhabha Atomic Research CentreTrombay, India

**Keywords:** transposable elements, plant stress tolerance, epigenetic, regulatory role, crop improvement

## Abstract

The recognition of a positive correlation between organism genome size with its transposable element (TE) content, represents a key discovery of the field of genome biology. Considerable evidence accumulated since then suggests the involvement of TEs in genome structure, evolution and function. The global genome reorganization brought about by transposon activity might play an adaptive/regulatory role in the host response to environmental challenges, reminiscent of McClintock's original ‘Controlling Element’ hypothesis. This regulatory aspect of TEs is also garnering support in light of the recent evidences, which project TEs as “distributed genomic control modules.” According to this view, TEs are capable of actively reprogramming host genes circuits and ultimately fine-tuning the host response to specific environmental stimuli. Moreover, the stress-induced changes in epigenetic status of TE activity may allow TEs to propagate their stress responsive elements to host genes; the resulting genome fluidity can permit phenotypic plasticity and adaptation to stress. Given their predominating presence in the plant genomes, nested organization in the genic regions and potential regulatory role in stress response, TEs hold unexplored potential for crop improvement programs. This review intends to present the current information about the roles played by TEs in plant genome organization, evolution, and function and highlight the regulatory mechanisms in plant stress responses. We will also briefly discuss the connection between TE activity, host epigenetic response and phenotypic plasticity as a critical link for traversing the translational bridge from a purely basic study of TEs, to the applied field of stress adaptation and crop improvement.

## Introduction

The solution of the *C*-value paradox, a well-known enigma dating from the earliest days of genome biology, led to the recognition of repetitive DNA, while simultaneously raising questions regarding its nature and possible function (Ohno, 1972; Doolittle and Sapienza, 1980; Orgel and Crick, 1980). Within the repetitive DNA, which constitutes the bulk of most eukaryotic genomes, transposable elements account for the major part. Furthermore, there exists a positive correlation of TE content with the organism genome size, though the individual TE classes vary as regards their presence in the respective genome (El Baidouri et al., 2014). TEs have been widely regarded as genomic parasites, with a tendency to self-perpetuate at the expense of host genomic stability (Doolittle and Sapienza, 1980; Orgel and Crick, 1980). However, systematic analysis across different plant systems has revealed their potential role in regulating host gene expression and genomic rearrangements (Lisch, 2012). The prevailing view considers the evolution of epigenetic processes as a defense against TEs and viruses, and TE involvement in any essential and adaptive host functions as matter of mutual coadaptation on part of the host and the TE (Kidwell and Lisch, 1997; Slotkin and Martienssen, 2007). However, this viewpoint is now being challenged in favor of TEs as the agents of evolutionary innovation and phenotypic plasticity (Fedoroff, 2012).

Initial clues regarding the nature of transposable elements came from a series of breeding experiments in maize, centered on a breakage region associated with variegated kernel color (McClintock, 1950). Unable to map these regions, McClintock surmised that these elements were in fact “mobile,” contributing to host genome dynamics. The Ac/Ds system thus opened a whole new dimension in the field of genetics, challenging the idea of a static genome with a more dynamic outlook. Since then, the overwhelming evidence accumulated via of a series of diverse studies has recognized these selfish DNA as useful parasites instead (Magalhaes et al., 2007; Hilbricht et al., 2008; Hayashi and Yoshida, 2009; Studer et al., 2011; Butelli et al., 2012; Guan et al., 2014; Li et al., 2014; Mao et al., 2015). As the evidence of their involvement in host genome evolution and function grows, one part of their role continues to be highlighted, i.e., in plant stress response to environmental challenges (Johns et al., 1985; Hirochika, 1993; Hirochika et al., 1996; Grandbastien et al., 1997; Bui and Grandbastien, 2012). Although TE activation under stress is considered a consequence of epigenetic deregulation (Slotkin and Martienssen, 2007), certain studies have hinted at a regulatory role for TEs, echoing the original controlling element hypothesis (McClintock, 1984; Bui and Grandbastien, 2012; Lisch, 2012). In this review, we attempt to explore these regulatory themes and associated applied aspects.

## Transposable elements (TEs): classification and organization in host genome

**Figure 1:**
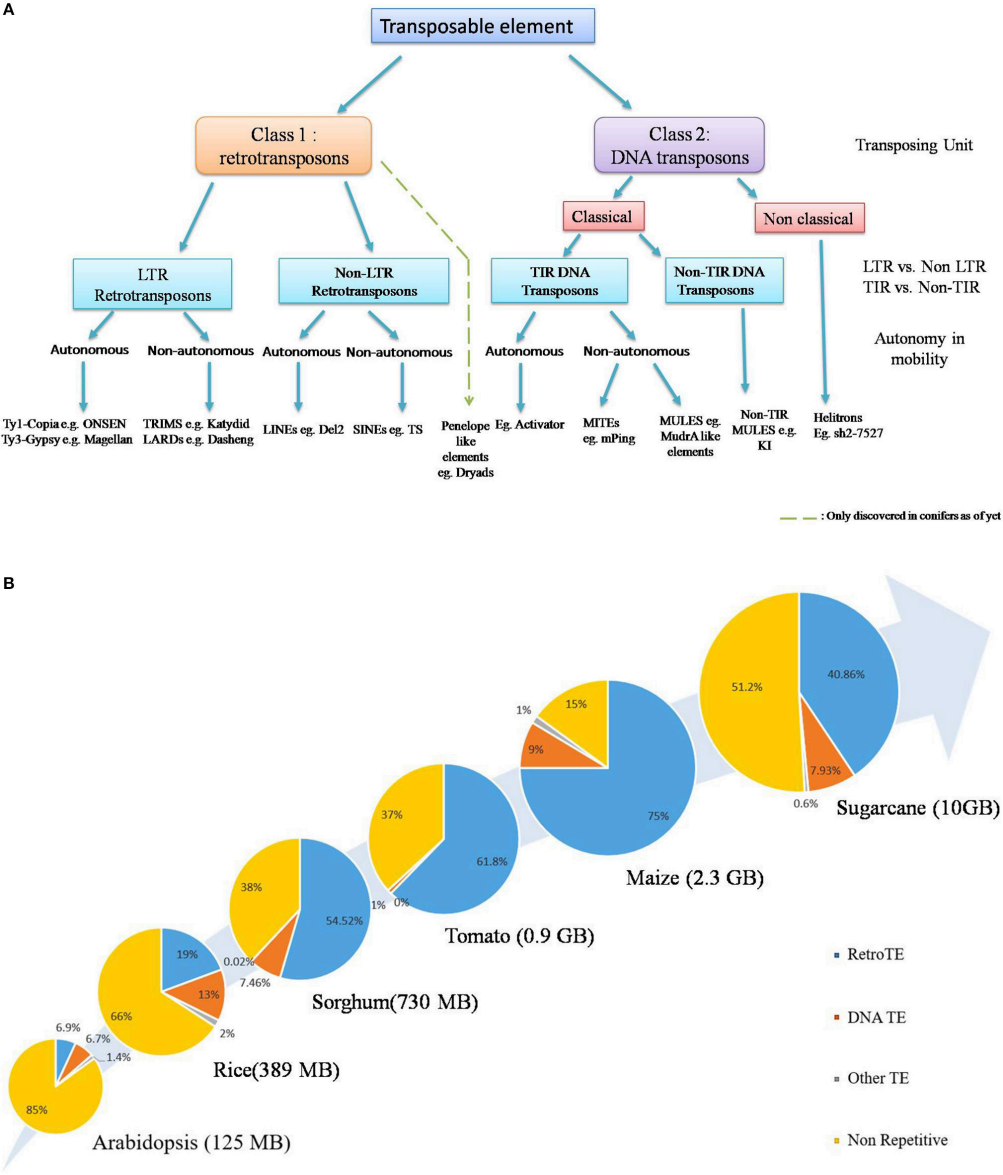
**(A)** General Classification of currently known plant transposable elements. According to the broadest classification system, TEs are divided into two classes on the basis of respective transposing unit: retrotransposons (class 1) and DNA transposons (class 2). Penelope like elements form a distinct group (Gladyshev and Arkhipova, 2007) and have been described only recently in conifers. Further classification is based on the classical /non classical mode of transposition, and the presence /absence of LTR, TIR sequences, though the phylogenetic origin of TEs is quickly gaining prominence as a key criterion for TE systematic (Modified from Wicker et al., 2007). Due to the lack of sufficient information about Helitron transposition, non-autonomous elements have not been indicated here. The presence of certain non-classical TEs such as Mavericks/Polintons have not been conclusively shown in plant systems yet and is thus not included here, exception being the Dryad element recently discovered in conifers (Lin et al., 2016). **(B)** TEs are major determinants of plant genome size. TEs, especially retrotransposons constitute the predominating part of plant species with big genomes. General increase in genome- size is positively correlated with increase in TE content. This is particularly true for cereal crops, such as maize and sugarcane. Source: (Arabidopsis Genome Initiative, 2000; International Rice Genome Consortium, 2005; Paterson et al., 2009; Schnable et al., 2009; Tomato Genome Consortium, 2012; de Setta et al., 2014).

In general, TE classification is based on type of chromosomal movement: Conservative (cut and paste) or Replicative (copy and paste) and the nature of the transposing unit. Though attempts have been made to devise a unified system of classification which tries to combine both the phylogenetic and enzymatic aspects (Figure 1A, Wicker et al., 2007; Kejnovsky et al., 2012), classification becomes rather difficult in the lower orders and most likely will require rigorous analysis of the aforementioned aspects (Piégu et al., 2015). The first class, known as retrotransposons, consists of an RNA molecule as the transposing unit which encodes proteins structurally homologous to retroviral gag- and pol-encoded proteins, facilitating their reverse transcription and subsequent integration (Kejnovsky et al., 2012). Retrotransposons are the predominant TEs in larger plant genomes such as maize, wheat and sugarcane and are further divided into those flanked by long terminal repeat (LTR) and those devoid of them (Figure 1A). The LTR retroelements are further divided into two major groups: Ty1-copia (Pseudoviridae) and Ty3-gypsy (Metaviridae), both of which are widely distributed across angiosperms. The Class II elements, on the other hand, transpose via a DNA intermediate and possess terminal inverted repeats (TIRs), which serve as sites of excision and reintegration by an element-encoded transposase (Finnegan, 1989). Non-classical transposons include helitrons, which transpose via a rolling-circle mechanism with the aid of a RepHel (Replicase-Helicase) protein with both lication initiator and DNA helicase domains, plus occasionally a replication protein A like single-stranded DNA-binding protein (Kapitonov and Jurka, 2001; Lal et al., 2003). So far their insertion has been reported in maize, where it accounts for about 2% of the genome (Kapitonov and Jurka, 2001; Lal et al., 2003; Gupta et al., 2005), although these have been computationally predicted in *Arabidopsis*, Rice (Kapitonov and Jurka, 2001; Xiong et al., 2014), as well as other plant genomes, such as *Sorghum*, rapeseed, *Medicago*, and solanaceous crops (Xiong et al., 2014). A third group is the Penelope like Elements, which are considered sufficiently distinct from the two major TE classes (Gladyshev and Arkhipova, 2007) and have been found recently in conifers (Lin et al., 2016). Another non-canonical TE family, the DIRS-like elements (named after *Dictyostelium* intermediate repeat sequence), have still not been described in higher plants (Kejnovsky et al., 2012). Apart from the autonomous TEs, non-autonomous TEs such as TRIMs (Terminal-Repeat Retrotransposons In Miniature) and LARDs (Large Retrotransposon Derivative) have also been discovered (Witte et al., 2001; Jiang et al., 2002) which owe their mobility to trans-complimentation by their autonomous counterparts.

The whole genomic TE complement, also referred to as the mobilome, constitutes a hefty chunk of plant genomes. Retrotransposons, in particular, can be several MBs in size and are known to be present in very high copy numbers, for example, the barley BARE-1, and Maize Opie-1, Cinful-1 and Huck2 reach up to 20,000–200,000 copies (SanMiguel et al., 1996; Vicient et al., 1999; Meyers et al., 2001), a reflection of their replicative mechanism of transposition. In what can be considered a direct answer to *C*-value paradox, it is now recognized that the respective changes in transposon content, especially retrotransposons, constitute a major source of genome size variation (Figure 1B). This is particularly supported by insights from cereal crops such as maize and sugarcane, where they account for approximately 40–75% of their genome respectively (Schnable et al., 2009; de Setta et al., 2014). DNA transposons generally form a relatively small portion of mobilome in higher plant genomes. Noticeable exceptions are MITEs such as the rice mPing (Jiang et al., 2003; Nakazaki et al., 2003; Naito et al., 2009) and the maize mPIF element, whose copy number reaches upto, 6000 (Zhang et al., 2001). In case of rice, MITEs, such as Stowaway like elements account for almost 2% of the genome (Mao et al., 2000) Despite this, the small size of MITEs (200–500 kb) mean that their contribution to plant genome size is relatively minor (Lee and Kim, 2014). The non-LTR retrotransposons, also forma substantial part of certain plant genomes (Casacuberta and Santiago, 2003), examples being TS, a SINE with over 50,000 copies in tobacco (Yoshioka et al., 1993) and Del2, a LINE with upto 250,000 copies in *Lilium* (Leeton and Smyth, 1993). Despite the lack of autonomy, certain LARDs such as Dasheng have upto, 1000 copies in the maize genome (Jiang et al., 2002).

Another key aspect of transposon genomics is the organization of TEs within the genome. Retrotransposons, particularly members of Ty3/gypsy *and* Ty1/copia super families are known to be present in high copies in centromeric heterochromatic regions with relatively lower rates of recombination (Arabidopsis Genome Initiative, 2000; He and Dooner, 2009), but are also associated with genes (White et al., 1994). Insights from barley genome showed that the major chunk of mobilome and other repeat structures is situated in random BACs, but less so in the gene bearing BACs (International Barley Genome Sequencing Consortium, 2012). As with rice genome, MITE's organization differs from that of LTR retrotransposons in that these are preferentially located within euchromatic regions as nested insertions (Zhang et al., 2000; Jiang and Wessler, 2001; International Rice Genome Consortium, 2005). In maize some of these insertions produced novel transcripts through shuffling of different host exons, paving the way for the evolution of novel proteins (Morgante et al., 2005; Du et al., 2009).

## TEs as functional mobile traces in host genome

As per McClintock's original genome shock hypothesis, TEs when triggered can bring about large-scale chromosomal rearrangements which might collectively shape the stressed host genome and facilitate adaptive evolution, as demonstrated in cereal genomes (McClintock, 1984; SanMiguel and Bennetzen, 1998; Vicient and Schulman, 2002; Du et al., 2009; Yu et al., 2011; Barbaglia et al., 2012; Ben-David et al., 2013) as well as eudicot genomes such as tomato (Bolger et al., 2014). The extent of their pervasion is demonstrated by the fact that TEs can directly control plant gene expression through interruption of regulatory motifs or by insertion of new regulatory circuits (Feschotte, 2008; Bonchev and Parisod, 2013). Evidence accumulated till date shows that TE insertion can function at multiple levels to regulate host expression and bring about interesting phenotypes, such as abiotic stress tolerance, fruit color, and so on (Lisch, 2012).

While TE activity appears to be fairly stable in most experimental populations, certain genomic, and physiological stresses, such as regulatory derepression through mutations in regulatory components, hybridization, tissue culture, and abiotic and biotic stresses can lead to a hike in the transposon activity (Hirochika, 1993; Hirochika et al., 1996; Petit et al., 2010). These situations reflect the transient TE-induced spikes in mutation rates in natural populations such as barley (*Hordeum spontaneum*) and in some cultivars of rice (*Oryza* spp.) which have been reported as a common occurrence (Kalendar et al., 2000; Naito et al., 2006; Ungerer et al., 2006). Considering the fact that artificial populations experience none of the vast array of biotic and abiotic stress that natural population are faced with regularly, these TE bursts and associated rate of null mutations are more likely to be accurate (Lisch, 2012).

### TEs are potent mutagens of the plant genomes

TEs are potent mutagens and hence the most direct consequence of TE activity is the generation of loss-of-function mutants, exemplified by the famous wrinkled pea phenotype, resulting from the insertion of an Ac/Ds TE in the starch branching enzyme SBEI (Bhattacharyya et al., 1990). The TE-induced mutations are similar to other null mutations but for the rate at which they arise, being subject to the activity and copy number of the TE in question (Lisch, 2012). Apart from disrupting the function of host genes, TEs can also disrupt positive or negative regulatory regions, to bring about interesting new twists in host gene expression. This is exemplified by the maize *Vgt1*, a conserved non-coding sequence (CNS) CNS present roughly 70 kb upstream of a gene that encodes an AP2 transcription factor which is a negative regulator of flowering (Salvi et al., 2002). An insertion of a MITE into the conserved portion of *Vgt1* is tightly associated with flowering time variation in maize (Salvi et al., 2002). Similarly, in rapeseed, BrFT2, an ortholog of the *Arabidopsis* FLOWERING LOCUS T (FT) gene is involved in regulation of flowering time (Zhang et al., 2015). A loss of function allele was generated via insertion of a retrotransposon in the second intron of BrFT2 in one of the parental lines, disrupting its expression The RILs carrying only the mutated BrFT2 allele showed delayed flowering regardless of growing seasons when compared to RILs carrying the wild-type BrFT2 allele. The consequence of TE insertion in regulatory regions like enhancers or repressors, is also exemplified by the insertion of *Mutator* elements into a CNS present in the first intron of the *knotted1* gene in maize which leads to its ectopic expression (Salvi et al., 2007). Additional examples include the certain horticultural traits such as seedless apples (Yao et al., 2001) and flower-color variation in morning glory (Park et al., 2007).

### TEs can modulate native gene expression patterns

While TE insertion can functionally impair the host gene, regulation may also come in the form of upregulation/repression of the gene expression profile or altered tissue specificity. A classic example is the maize tb1, which encodes a transcription factor which represses branching (Studer et al., 2011). The presence of a retrotransposon, *Hopscotch*, present as far as 60 kb upstream of the tb1 locus, greatly enhances its expression resulting in a dramatic reduction in the number of branches relative to the progenitor species. This example clearly demonstrates how TE-induced changes in gene expression, are literally “far-reaching” and have been a shaping influence in the evolutionary domestication of maize (Studer et al., 2011; Lisch, 2012). The alteration in tissue specificity due to TE activity is exemplified by the pigmentation alleles in maize. The insertion of an active retroelement in the first exon of the maize b1 gene shifted the expression pattern from vegetative tissues to the seed, creating the B-Peru allele (Selinger and Chandler, 2001). Further transposon insertion in this locus, created the B-Bolivia allele, which further reduced the expression of b1 to yield a more variegated phenotype.

### TEs can introduce new genetic information

Additionally, TEs can also bring some novelty to the genome architecture to introduce new coding information. Many Transposon families (such as *Mutator*, Ac, and *mPing*) tend to insert into the 5′ region of genes, which, combined with the elemental information TEs carry, can give rise to interesting new patterns of gene expression (Pan et al., 2005; Naito et al., 2009; Vollbrecht et al., 2010; Lisch, 2012). In rice, a recent burst of mPing activity was detected (Naito et al., 2009). The comprehensive sequence analysis of 1664 mPing insertion sites in rice genome and their impact on the expression profile of 710 genes revealed a surprising avoidance of exon insertions and a preference for insertion into 5′ flanking regions instead. Moreover, an increase in expression was observed in case of 156 genes. Thus, TEs act as a natural reservoir of regulatory information for host gene-expression networks.

### TEs can move host genes

Gene movement is another mechanism by which transposons exert control over the host gene expression, as it transports host genes in an entirely new setting of regulatory regions, methylation backgrounds, and chromatin landscapes that might cause neofuntionalization or silencing, depending upon the altered genomic context (Lisch, 2012). This may constitute another mechanism by which TEs can act as agents of evolutionary evolution (Fedoroff, 2012; Lisch, 2012). The visibly phenotypic traits again provide an example, in this case the oval shape of Roma tomatoes (van der Knaap et al., 2004). Changes in fruit shapes between some varieties of *Solanum lycopersicum* and its round-fruited wild relative *Solanum pimpinellifolium* are largely caused by the variation at the *sun* locus. The movement of *IQ domain 12* (*IQD12*)- the key gene at the sun locus to *DEFL1*, a fruit specific gene causes *IQD12* to be expressed in fruit now, bringing about the round shape fruit which is typical of the Roma variety (van der Knaap et al., 2004).

### TEs affect epigenetic makeup of host loci

So far the prevailing view on epigenetic processes and TE amplification has been that the extensive epigenetic arsenal has evolved in plant systems to suppress TE bursts and such the TE activity observed under stress conditions is due to epigenetic deregulation instead (Slotkin and Martienssen, 2007). An alternate view which is now gaining prominence highlights TEs as the mediators of epigenetic responses instead (McClintock, 1984; Mirouze and Paszkowski, 2011; Fedoroff, 2012). Thus, along with the co-transcriptional regulation, the TE-facilitated post-transcriptional regulation of expression of neighboring gene, can effect far-reaching phenotypic outcomes. This is exemplified by the *FLOWERING WAGENINGEN* (*FWA*) locus in *A. thaliana* (Kinoshita et al., 2007). The presence of a SINE element immediately upstream of the *FWA* locus causes it to be epigenetically silenced in vegetative tissues by small RNA processing and DNA methylation. In mutants where the small RNA processing or DNA methylation can result in ectopic expression of *FWA*, a late flowering phenotype results, which manifests itself as a stable epimutation. In addition, allelic variation in FLOWERING LOCUS C (FLC), a central repressor of flowering, contributes to differences in flowering behavior among natural *Arabidopsis* accessions. The insertion of a 1.19 kb non-autonomous Mutator-like TE in first intron of FLC locus renders FLC-Ler (Landsberg Erecta) to siRNA mediated repressive chromatin modifications, reminiscent of the “controlling Element” hypothesis (McClintock, 1950; Liu et al., 2004). The consequent attenuation of FLC expression causes delayed flowering, which is speculated to provide a fitness advantage as the extension of vegetative development can lead to more robust plants with high seed yield (Strange et al., 2011).

### Exaptation of transposon sequences into host genes

From an evolutionary angle, the co-option of adaptive features naturally selected for one role, for a new role is called exaptation. Concurrent with this viewpoint, TE sequences, such as exons and binding sites can be directly exapted for specific phenotypic functions in the organism (Hoen and Bureau, 2012). There are multiple examples of TE sequence exaptation in animal systems (Volff, 2006). In plants fewer such examples exist, yet they are critical to plant growth and development. These include FAR1 and FHY3, two transcription factors involved in phy-A signaling, domesticated from the maize TE Jittery (Lin et al., 2007), Mustang(MUG1-8) derived from the MULE superfamily which plays an important role in flower development and reproductive fitness (Cowan et al., 2005; Joly-Lopez et al., 2012), the *Arabidopsis* DAY SLEEPER, important in plant body development (Bundock and Hooykaas, 2005) and the barley Garyand sugarcane SchAT, members of hAT transposase-like gene family (Muehlbauer et al., 2006; Sinzelle et al., 2009; de Jesus et al., 2012).

### Transduplication: host gene capture by moving TEs

Further mechanisms by which transposons can modulate the host transcriptional machinery are transduplication, which has the potential to create new genes. This mechanism holds holds special significance in case of maize, where 2791 gene fragments are known to be captured by *Helitron* elements in the past (Lal and Hannah, 2005; Du et al., 2009; Dong et al., 2011). While many of these gene fragments might be non-functional pseudogenes, some of these may be expressed, generating potential for neofunctionalization. In addition, Pack-MULE has been known to acquire host genes on frequent basis (Jiang et al., 2004; Juretic et al., 2005). As sufficient data on the evolutionary selection of these new gene is not available, further analysis of such gene capture events would be required to establish the functionality of these new genes unambiguously (Lisch, 2012).

## *Two to tango*: plant stress and transposons

Given the sessile habit of plant systems, the phenomenon of environmental stress, and the corresponding response holds a special significance. As stress impacts the plant at various levels, the plant response to it also manifests at multiple levels of organization (molecular, tissue, anatomical, and morphological), such as the adjustment of the membrane system, alterations in cell wall architecture, transcriptional, and metabolic response (Shinozaki et al., 2003; Hirayama and Shinozaki, 2010). Through the history of evolution, plants have amassed an impressive array of sophisticated response machinery: sensors, signaling components, regulatory components, transcription factors, and effectors that function to “sense, respond, and adapt”- working in an orchestrated fashion to bring about a multifarious phenotypic response (Shinozaki et al., 2003; Hirayama and Shinozaki, 2010). It has also become evident that different stress response pathways intersect at many points and their crosstalk gives way to an intricate network which constitutes not only the immediate component of stress alleviation, but also possibly of memory (Bruce et al., 2007; Walter et al., 2013; Kinoshita and Seki, 2014; Avramova, 2015).

The activation of transposons under plant stress is a well-known phenomenon (Johns et al., 1985; Hirochika, 1993; Hirochika et al., 1996; Turcich et al., 1996; Grandbastien et al., 1997; Bui and Grandbastien, 2012). The first instance of a TEs being induced by stress was the maize Bs1, in response to barley stripe mosaic virus infection (Johns et al., 1985). Since then, several studies have revealed the activation and/or transposition of TEs in response to a diverse array of stresses (Table 1). Abiotic (irradiation, temperature) and biotic (culture tissues or infections by viruses or pathogens) stresses are known to awaken quiescent TEs in plants (Grandbastien et al., 2005; Bouvet et al., 2008). Irradiation is known to induce biological events like mutagenesis and genomic instability. Activation of several retrotransposons has been shown which is variable and subject to radiation type. For example, ion beam, a type of high linear energy transfer (LET) radiation causes relatively few but large, irreparable DNA lesions (Tanaka, 1999). Hence, it is expected that dormant transposons may be induced by ion beam irradiation in rice. UV-B caused activation of cryptic TEs could also increase the mutation rate (Walbot, 1999). Gamma irradiation impact on transposon activation has been also shown in yeast, where it acts on multiple levels in Ty1 life cycle.Ty1 transcription and transposition is repressed in diploid cells and ste12 mutants, where both yet gamma-irradiation affects both an increase in Ty1 RNA levels as well as its transposition (Sacerdot et al., 2005). Interestingly, the stress induced retrotransposon response has been shown to be genotype specific (Ansari et al., 2007; Long et al., 2009; Lopes et al., 2013; Berg et al., 2015).

**Table 1:** **Selected examples demonstrating stress-induced activation of transposable elements under various environmental challenges**.

**TE**	**Plant system**	**Tissue/Organ**	**Stress/Environmental stimulus**	**Transcriptional regulation and/or transposition**	**Methodology**	**References**
**MITE**						
*mPing*	*Oryza sativa*	Leaves of callus regenerants	Cell culture	Transposition	Transposon display	Ngezahayo et al., 2009
	*Oryza sativa*	Leaves	Inter-specific hybridization	Transposition	Locus excision display, Southern blotting	Wang et al., 2009
	*Oryza sativa*	Leaves of M0 generation	Gamma Irradiation	Transposition	Transposon display	Nakazaki et al., 2003
	*Oryza sativa*	Anther-derived calli	Anther culture	Transposition	Southern Hybridization	Kikuchi et al., 2003
	*Oryza sativa*	Leaves of introgresses lines	interspecific hybridization	Transposition	Locus excision display, Transposon display	Shan et al., 2005
	*Oryza sativa*	Leaves	Hydrostatic pressurization	Transposition	Locus excision display, Transposon display	Lin et al., 2006
*mGing*	*Oryza sativa*	M1 generation seedlings, Callus regenerants, Scutellum-derived calli	high dose γ-ray irradiation, anther and scutellum callus culture	Transposition	Transposon display	Dong et al., 2012
*ZmTPApong*	*Zea mays*	Anther derived calli	Cell culture	Transcriptionally activation: cell culture and Transposition: regenerated progeny	cDNA-AFLP	Barret et al., 2006
Minos	*Triticum aestivum*	Leaves	interspecific hybridization	Transposition	454 Pyrosequencing, Excision assay, transposon display	Yaakov et al., 2012
**DNA TRANSPOSON**						
TCUP	*Zea mays*	Leaves	long-term tissue culture and azacytidine treatment	Transposition, Transcriptional upregulation	Transposon display, RT-PCR	Smith et al., 2012
Pong	*Oryza sativa*	Leaves of introgressed lines	interspecific hybridization	Transposition	Locus excision display, Transposon display	Shan et al., 2005
	*Oryza sativa*	Leaves	Hydrostatic pressurization	Transposition	Locus excision display, Transposon display	Lin et al., 2006
*Ping*	*Oryza sativa*	Leaves of M0 generation	Gamma Irradiation	Transposition	Transposon display	Nakazaki et al., 2003
*Mx, PIF*	*Zea mays*	Cultured cells	cell culture	Transcript abundance	EST library	Vicient, 2010
*Tam3*	*Antirrhinum majus*	Low temperature		Transpostion	Locus excision display	Uchiyama et al., 2008
**RETROTRANSPOSONS**						
*Athila*	*Arabidopsis thaliana*	Suspension cultures	geminivirus proteins treatment	Trancriptional upregulation	Northern blotting, and RT-PCR	Buchmann et al., 2009
			prolonged heat stress	Transcriptional upregulation	Quantitative PCR	Pecinka et al., 2010
*TLC1/Retrolyc1*	*Solanum chilense*	Leaves	wounding, ethylene, auxins, abscisic acid, jasmonate, salicylic acid, H_2_O_2_	Transcriptional upregulation	Northern blotting, RT-PCR	Tapia, 2005
*Osr7, Tos17 and Osr23*	*Oryza sativa*	Leaves	Inter-specific hybridization	Transposition	Southern blotting	Wang et al., 2009
TLC1. 1	*Solanum chilense*	Leaves	alicylic acid, abscisic acid, methyl jasmonate, hydrogen peroxide and 2,4-D	Transcriptional upregulation	RT-PCR, Promoter:GUS Fusion	Salazar et al., 2007
*HRET1*	*Hibiscus syriacus*	Leaves	wounding	Transcriptional upregulation	Northern Blotting	Jeung et al., 2005
*LORE1*	*Lotus japonicus*	Progenies of callus-regenerants	Callus culture	Transposition and transcriptional upregulation	SSAP, Quantitative PCR	Madsen et al., 2005
		Pollen of callus-regenerants	Callus culture	Transcriptional upregulation	RT-PCR	Fukai et al., 2010
*Hopscotch-like*	*Saccharum officinarum*	Callus	Callus culture	Transcription upregulation	EST library	de Araujo et al., 2005
*Morgane*	*Triticum aestivum*	Leaves	fungal infection various biotic/abiotic stresses and nitrogen stress	Presence in EST abundance	EST library	Sabot et al., 2006
*CCR*	Oryza sativa	Aerial tissue	Callus, drought, cold and fungal inoculation	Increased EST abundance	RT-PCR, Northen blot	Neumann et al., 2007
*CIRE1*	*Citrus sinensis*	Leaves	Wounding and auxin treatment	Transcriptional upregulation	Semi quantitative PCR	Rico-Cabanas and Martínez-Izquierdo, 2007
Various	*Oryza sativa*	Aerial tissue, suspension culture	Cell culture, drought and salt stress	Transcription upregulation	Microarray	Jiao and Deng, 2007
*Erika, Romani*	*Triticum aestivum*	Roots	Fungal inoculation and mycotoxin treatment	Genotype-dependant transcriptional upregulation	differential display analysis and RT-PCR	Ansari et al., 2007
BAGY1	*Hordeum vulgare*	Leaves	Senesense	Transcription upregulation	RT-PCR	Ay et al., 2008
*AtCopeg1*	*Arabidopsis thaliana*	Whole seedling	Nutrition starvation, salt stress, cytokinine, abscisic acid	Upregulation: nutrition starvation, salt stress and cytokinine, downregulation: abscisic acid		Duan et al., 2008
*LORE2*	*Lotus japonicus*	Leaves	Cell culture	Transcriptional upregulation and transposition	Southern and northern blotting	Fukai et al., 2008
*Reme1*	*Cucumis melo*	Leaves	UV light	Transcriptional upregulation	RT-PCR	Ramallo et al., 2007
*CLCoy1*	*Citrus lemon*	Leaves	Wounding and salt stress, cell culture	Transcriptional upregulation	RT-PCR, Quantitative PCR	Felice et al., 2009
Various	*Vitis vinifera*	Berry fruit	Post-harvest withering (dehydration)	Transcriptional upregulation	AFLP-TP analysis	Zamboni et al., 2008
*MERE1*	*Medicago truncatula*	Calli, callus regenerated plants	Tissue culture	Upregulation: calli and transposition: callus-regenerated plants	RT- PCR, Transposon Display	Rakocevic et al., 2009
*Osr23, Osr36, Osr42*	*Oryza sativa*	Leaves	Spaceflight	Genotype-dependent transpositional activation	Southern blotting	Long et al., 2009
*FaRE1*	*Fragaria x ananassa*	Leaves	Auxins and abscisic acid treatment	Transcriptional upregulation	RT-PCR and northern blotting	He et al., 2009
*At2G06045*	*Arabidopsis thaliana*	Whole seedling	Salt, osmotic, cold and heat stress abscisic acid (ABA) treatment	Salt, cold, osmotic stress and ABA: transcriptional upregulation and heat stress: transcriptional downregulation	whole-genome tiling arrays	Zeller et al., 2009
*Ttd1a*	*Triticum durum*		Salt and light stress	Transcriptional upregulation and transposition	Semi quantitative PCR and SSAP profilling	Woodrow et al., 2010
Various	*Lolium perenne*	Pseudostem tissue	Pathogenic fungal interaction	Interaction dependant Up- or downregulation	RNA Seq	Eaton et al., 2010
*ONSEN/Atcopia78*	*Arabidopsis thaliana*	Whole plant	Prolonged heat stress	Transcriptional upregulation	cDNA microarray	Pecinka et al., 2010
		Whole seedling	Induced temperature stress	Transcriptional upregulation	cDNA microarray	Tittel-Elmer et al., 2010
		Callus	Heat stress	Transcriptional upregulation and Transposition	Quantitative PCR and Southern Blotting	Matsunaga et al., 2012
		Progeny of stressed mutants	Heat stress	Transcriptional transposition	Northern blotting and transposon display	Ito et al., 2011
		Leaves	Elicitation with bacterial flagellin	Transcriptional upregulation	Quantitative PCR	Yu et al., 2013
Various	*Arabidopsis thaliana*	Whole seedlings	Cold-heat temperature shifts	Transient upregulation	cDNA microarray	Tittel-Elmer et al., 2010
Various	*Arabidopsis thaliana*	Whole plant	Prolonged heat stress	Transcriptional upregulation	cDNA microarray	Pecinka et al., 2010
Various	*Zea mays*	seedlings	Water deficit	Transcriptional Up- or down-regulation	cDNA microarray	Lu et al., 2011
Various Retro- and DNA transposons	*Oryza sativa*	Roots and shoots	Atrazine treatment	Element specific- transcriptional Up or downregulation	RNA Seq	Zhang et al., 2012
*Tcs1, Tcs2*	*Citrus sinensis*	Fruit juice	Cold stress	Transcriptional upregulation	Quantitative PCR	Butelli et al., 2012
*Corky*	*Quercus suber*	Leaves	Wounding	Transcriptional upregulation	Quantitative PCR	Rocheta et al., 2012
*FIDEL*	*Arachis spp*	Leaves and roots	Infection with *C. Personatum* and gradual drought stress	Transcritional upregulation	RNA Seq	Guimarγes et al., 2012
Various	*Coffea arabica*	Leaves	Drought stress	Genotype-specific response	Macroarray	Lopes et al., 2013
*AtGP1, EVD*	*Arabidopsis thaliana*	Leaves	Elicitation with bacterial flagellin	Transcriptional upregulation	Quantitative PCR	Yu et al., 2013
Various	*Arabidopsis thaliana*		Arsenic stress and phosphate starvation	transcriptional upregulation	Microarray	Castrillo et al., 2013
Various MITEs	*Oryza sativa*	Early microspore stage anthers	Cold stress	transcriptional up or downregulation depending on the genotype	Microarray	Ishiguro et al., 2014
Various retrotransposons	*Pinus sylvestris*		Heat stress, aphids infestation, and abscisic acid salicylic acid treatment.	transcriptional upregulation	iPBS	Voronova et al., 2014
*Queenti*	*Nicotiana tabacum*	Cell suspentions, leaves	Fungal elicitin-Cryptogein treatment, ROS and H2O2	Upregulation byCryptogein, downregulation by ROS and H2O2	Semi quantitative and Quantitative PCR	Anca et al., 2014
***SINES***						
Alu- SINEs	*Triticum aestivum*	Leaves	Inter-specific hybridization	Transposition	site-specific PCR and transposon display	Ben-David et al., 2013

The global upregulation of transposon activity in response to stress is a well-established fact. The “Controlling Element” hypothesis placed a special emphasis on their potential role as genomic modulescontrolling host gene expression (Bui and Grandbastien, 2012). In McClintock own words “The real point is control. The real secret of all of this is control. It is not transposition.” According to this hypothesis, environmental stress boosts TE expression and transpositional activity, leading to extensive genomic changes that facilitate the adaptation of populations and species facing changing environments (McClintock, 1984). The coevolutionary angle on these transpositional bursts suggested that while such bursts of activity, is self-perpetuating in intent, it might bring about genetic variation on a global scale. For the host, this extensive genome restructuring facilitates stress adaptation and ultimately evolution (Kidwell and Lisch, 1997). However, a more regulatory view of TEs is garnering support in light of recent evidences which establish these as “distributed genomic control modules” (Bui and Grandbastien, 2012; Fedoroff, 2012). The manner in which the TEs participate in this could also be different. The TE occupying heterochromatic regions e.g., the LTR retrotransposons, by virtue of their size, stress responsive promoters, and replicative mechanism of transposition, could sense the physiological or environmental cues affecting alterations in the overall genome architecture (Kidwell and Lisch, 1997). The TEs closely associated with genic regions could be involved in actively reprogramming of host transcriptional networks, affecting specific expression characteristics of individual genes ultimately fine-tuning the host response to specific stimuli (Shapiro, 2005; Lisch, 2012).

### Transposing toward a regulatory role

**Figure 2:**
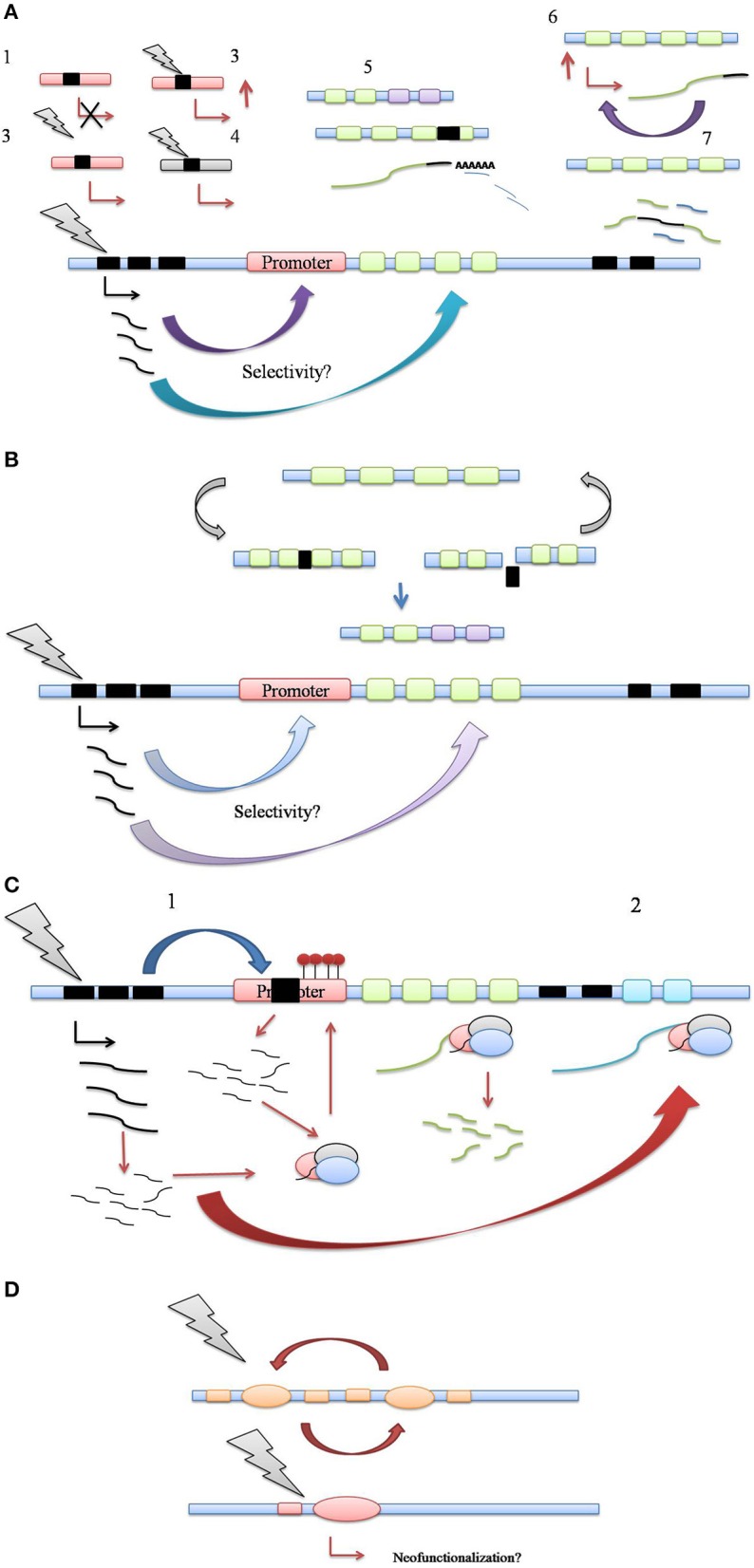
**(A)** Control of host stress response genes via transposition in regulatory and coding regions. (1) TE insertion into promoter can simply lead to loss of promoter function, keeping with their mutagenic activity, (2) TE insertion can enhance host gene expression or alter gene expression patterns, such as tissue specificity, (3) TE insertion confers stress-responsiveness to host promoter through its own cis-elements, (4) TE insertion may refunctionalise defunct host gene promoters, (5) TE insertion may lead to indel mutations in exonic regions, altering polyadenylation patterns of host gene transcript, regulating its expression through transcript abundance, (6) TE insertion into 3′ UTR might increase the expression and/or stability of target gene transcript, leading to increased transcript abundance, (7) Aberrant splicing of target gene transcript through TE insertion in intronic regions may lead to generation of novel gene combinations. **(B)** TE driven genetic polymorphism in plant defense response. The genomic regions housing defense genes clusters also contain TE clusters. Multiple cycles of TE insertion and excision into coding regions of defense genes facilitate their allelic recombination. Resulting allelic polymorphism may prove crucial in plant's molecular arms race with newly evolving pathogen races. **(C)** Control of host stress-response genes via generation of small noncoding RNA. (1) TE insertion into promoter regions may cause the silencing of host gene expression via the generation of regulatory small RNA generation and RdDM pathway, (2) siRNA generation and Ago complex mediated processing of target host transcript via 3′ UTR targeting might lead to post-transcriptional gene silencing. **(D)** Recruitment of TE coding sequences as host stress-response genes. Through multiple cycles of insertion/excision, TE coding sequences such as transposase can be exapted as host genes, leading to a scope for new functionality. Thus, TEs can serve as the reservoir of not just regulatory but also coding information for evolution of host genes, including those involved in stress response.

While aforementioned studies staunchly prove the activation of TE under various environmental challenges, solid evidence for a regulatory role of TEs in host stress adaptation is still scarce. A series of recent studies have pointed out this facet of transposon activity, highlighting their potential role as regulators of host gene expression for host stress amelioration (Magalhaes et al., 2007; Hilbricht et al., 2008; Hayashi and Yoshida, 2009; Bui and Grandbastien, 2012; Butelli et al., 2012; Tsuchiya and Eulgem, 2013; Guan et al., 2014; Li et al., 2014; Mao et al., 2015). Based on the studies certain common mechanistic themes have emerged which explain most of the naturally present transposon modulation of host stress responses (Figure 2).

### TE insertion into regulatory regions facilitates stress inducible gene expression

Viewed mechanistically, the inherently mobile nature of transposons suggests movement into the genic regions as an obvious mechanism of gene regulation. One of the most striking examples of transposons acting as a regulatory element under stress is the blood orange fruit trait, a consequence of the LTR-driven transcriptional activation of the Ruby Myb gene, an activator of anthocyanin synthesis (Butelli et al., 2012). The Ruby locus is inactive in naval blond oranges, but the insertion of the Tcs1 retrotransposon in its promoter, drives its expression in a cold-dependent fashion in Sicilian blood oranges, whereupon the Tcs1 3′ LTR provides transcriptional start and regulation. Furthermore, in some Sicilian blood orange accessions Tcs1 has undergone recombination to form a solo-LTR, sufficient to maintain Ruby expression. Interestingly in Chinese blood orange variety, there exists an upstream insertion of Tsc2, another copy of the same retrotransposon, however in reverse orientation to Ruby, suggesting that regulatory motifs have been preserved either in the U3 or the U5. This study demonstrates how two parallel, yet independent, LTR recruitments influence the host gene expression to perform similar functions, despite the possibly artificial selection of blood orange phenotype (Butelli et al., 2012; Lisch, 2012). It was speculated that the observation of the cold dependence of this desired phenotype led to the artificial implementation of cold in the early selection steps, which could have mobilized this particular retrotransposon (Bui and Grandbastien, 2012; Lisch, 2012).

In rice, the GSTL2 promoter further demonstrates the effect of transposon insertion on host stress responses (Hu et al., 2011). The plant glutathione S-transferase genes play an important in herbicide detoxification. The characterization of its promoter region through deletion analysis revealed that it contains two transposons; a partial Ds-like element and a stowaway-like element resulting in a reduced expression from this promoter, but this reduction was balanced by the presence of enhancer elements (Hu et al., 2011). Moreover, the study suggested that the original promoter of this gene was constitutive in expression, and the cis-elements responsible for its hormone and herbicide responsible expression might have been attributed by the TEs instead (Hu et al., 2011). In rice, the differential transcriptomic analysis of rice seedlings exposed to iron excess revealed a large no. of LTR retrotransposons to be upregulated. While no direct role of transposons in iron excess-responsive gene upregulation could be established, the authors found certain common cis regulatory elements in the promoters of upregulated host genes and LTRs of retrotransposons, which warrants further investigation (Finatto et al., 2015).

TE insertion in other regulatory regions also brings about stress inducible expression of associated genes. A haplotype study conducted in wheat (*Triticum aestivum*) comparing the expression of TaHSP16.9-3A in a heat-tolerant and susceptible cultivar (TAM107, and Chinese Spring respectively), showed its enhanced expression in TAM107 under heat stress (Li et al., 2014). The heightened expression of the TaHSP16.9-3A was found to be due to the presence of a tourist-like MITE into its 3′ UTR, which enhanced its transcription. Remarkably, the effect of MITE insertion and the subsequent higher expression was consistent throughout several wheat varieties which possessed the mutant haplotype. Although the 3′ UTR is well-known as the determinant of mRNA stability, this study suggested a possible cis-element like role for MITEs in heat stress responsive enhancement of HSP expression (Li et al., 2014).

A key insight in TE activity with host epigenetic processes in context of plant stress is provided by ONSEN, in *Arabidopsis thaliana*. ONSEN is an Copia-type LTR retrotransposon that is transcriptionally activated under heat stress, whose transposition in *Arabidopsis* involves an epigenetic mechanism (Ito et al., 2011; Matsunaga et al., 2012). Transposition of ONSEN is more frequent in the RdDM complex mutants exposed to heat stress, suggesting a role for RdDM machinery in the prevention of transgenerational propagation of ONSEN (Ito et al., 2011). The newly synthesized ONSEN copies decay post stress, suggesting that the stress-induced transcriptional activation of ONSEN may trigger a feedback mechanism that re-establishes methylation and silencing of TEs following recovery from stress. Despite the post-stress decay of newly synthesized transcripts, a burst in transposition was detected in the progeny of the progeny of the RdDM complex mutants. This was attributed to the persisting memory of stress, as a consequence of the faulty epigenetic reponse in these mutants (Ito et al., 2011). An *Arabidopsis* locus with ONSEN insertion in Col-O ecotype was known to be heat responsive (Lim et al., 2006) but the transcriptional response of the same locus in ecotype Zurich, devoid of ONSEN insertion, was considerably low (Ito et al., 2011), suggesting the genotype specificity generally observed in TE responses (Ansari et al., 2007; Long et al., 2009; Lopes et al., 2013; Berg et al., 2015). Through an artificial system designed to induce experimental bursts of ONSEN in a controlled fashion it was observed that even the loci affected by such stress-induced TE bursts acquired heat responsiveness (Ito et al., 2011). The promoter regions of ONSEN contains a heat shock element (HSE) shown to bind host heat shock transcription factors and permitting its heat inducible expression (Cavrak et al., 2014). This example successfully demonstrates a possible mechanism by which a TE can modulate the host transcriptional circuits in response to stress (Ito et al., 2011; Fedoroff, 2012).

### TE generated mutant alleles in host stress response

TEs are potent mutagens, and create definite genetic variations at the insertion loci, which could be selectively neutral, fatal, or beneficial for the host. The tight evolutionary pressure governing plant genomes ensures the removal of lethal insertions by failure of survival, the neural and beneficial ones may get selected and fixed in the host genome. The potential role of TEs as regulators of host gene expression is particularly supported by data from the rice genome. Nearly one-sixth of rice genes are associated with retrotransposons (Krom et al., 2008), whereas 58% are associated with a MITE (Lu et al., 2012), suggesting that a large proportion of rice gene promoters appear to contain a TE. For instance, a gene called *Pit* confers resistance to rice blast disease in the cultivar K29 (Hayashi and Yoshida, 2009). The comparative sequence analysis of the Pit allele between K29 and a susceptible cultivar, Nipponbare revealed that the Pit allele in K29 contains both a DNA transposon *dDart*, and a long terminal repeat (LTR)-retrotransposon, named *Renovator*. These results, combined with transgenic studies chiefly attributed the resistance phenotype to the presence of the LTR retroelement (Hayashi and Yoshida, 2009). A similar study was recently made in cucumber, where the locus in question, CsaMLO8, is a candidate powdery mildew susceptibility gene residing in the QTL conferring hypocotyl resistance (Berg et al., 2015). The comparison of alleles of CsaMLO8 from resistance and susceptible genotypes showed the presence of 2 non frameshift deletions of 172 and 74 bp in case of resistant genotypes. These deletions result from aberrant splicing of the CsaMLO8 transcript caused by the presence of a non-autonomous LTR retroelement, leading to the complete and partial loss of the exon 11, which is highly conserved among the susceptible genotype. Similar findings have been reported in pea (Humphry et al., 2011).

Metal tolerance is a root associated trait and a recent study revealed TE insertion as a contributory factor. In sorghum, the principal aluminum tolerance locus in sorghum is *AltSB*, which encodes three ORFs, out of which only one-a MATE (multidrug and toxic compound extrusion), shows high enough expression in roots where Aluminium quenching occurs (Magalhaes et al., 2007). SbMATE is an aluminum responsive citrate transporter whose coding sequence is the same in both the Aluminium tolerant and susceptible cultivars, the only significant variation being the presence of a Tourist—like MITE which confers root specific expression to this transporter (Magalhaes et al., 2007). In rice, stress activated TEs have been showed to be involved in host response to cadmium toxicity (Ishikawa et al., 2012). Using accelerated carbon atoms, Ishikawa and coworkers recovered three mutants showing drastic reduction in accumulated grain cadmium levels. They mapped these changes to a region housing two putative heavy-metal transporters. Sequence comparison with the WT loci indicated OsNRAMP5 as the center of these changes. While all the three lines experienced mutation in this region, one line-lcd-kmt1, had a 433-bp insertion identical to mPingA1 in the exon X replacing the terminal 32 bp, the remaining insertion being spliced out with intron X. The disruption of this cadmium transporter caused a significant reduction in grain cadmium levels, without much change in the agro-morphological quality characteristics of the parent variety (Ishikawa et al., 2012).

The deleterious effect of TE insertion in host stress genes have also been demonstrated in soybean (Guan et al., 2014). Using a fine mapping approach in the population derived from commercial cultivars Tiefeng 8 and 85–140 (salt-tolerant and salt-sensitive parent respectively), Guan and coworkers identified GmSALT3 (salt tolerance-associated gene on chromosome 3), a major gene encoding an ER-localized cation/H^+^ exchanger protein that contributes to a salt tolerance phenotype through sodium exclusion in shoots (Guan et al., 2014). They showed that the disruption of the third exon by a 3.78-kb Copia-like retrotransposon, produced a truncated transcript contributed to salt-sensitivity in the cultivar 85–140 (Guan et al., 2014). Further attesting to the capability of TEs to rewire innate transcriptional circuits is the *ArabidopsisCOPIA-R7*. Inserted in the first intron of *RPP7* (Resistance to *Peronospora parasitica 7*), COPIA-R7 introduces an alternative polyadenylation site; the critical balance between the functional and nonfunctional RPP7 then decides the host responsiveness to the pathogen transcripts (Tsuchiya and Eulgem, 2013).

### TE driven genetic polymorphism in plant defense

The plant resistance genes are known to be subject to high levels of polymorphism and are subject to adaptive evolution. For example, the rice Xa21 gene family houses as many as 17 TEs (Song et al., 1997, 1998); even a closely linked marker pTA818 encodes a transposon protein (He et al., 2000). Moreover, some of these insertions have given rise to novel proteins upon insertion. This is demonstrated by Xa21D, which shows 99.9% similarity to the resistance conferring allele, before and after a Retrofit insertion, but encodes a truncated protein lacks the transmembrane or kinase domain present in its progenitor. Furthermore, TE insertion may arise spontaneously in these resistance genes, such the *dLute* insertions in L6 flax rust resistance gene (Luck et al., 1998). Their insertion and subsequent reversion or imprecise excision serves as an evolutionary device in the generation of high allelic variability, a fundamental prerequisite for emergence of new pathogen race specificities (Richter and Ronald, 2000). One such example is the evolution of the ALP-A3 gene in diploid wheat (Akhunov et al., 2007). The original progenitor, ALP-A1 specifies an acireductonedioxygenase-like protein, and has undergone duplication, with the new duplicated gene acting as the parent of the next duplication. ALP-A3, while having the complete coding region, has seemingly lost the promoter region, instead being driven by a promoter sequence originating from a CACTA element (Akhunov et al., 2007).

### Source of small RNA regulating plant abiotic stress response

Almost 23.5% of all small RNAs identified from rice are derived from MITEs (Lu et al., 2012). TE mediated regulation of plant stress-responsive gene expression also occurs due to the generation of small non-coding RNA that originate from TEs in response to stress. This is exemplified by the generation of a cluster of small RNAs (smRPi1LTR) derived from the Copia95 retrotransposon LTR region under phosphate (Pi) starvation, in *Arabidopsis* (Hsieh et al., 2009). A comparative analysis of the expression pattern between Columbia and Landsberg accessions indicated that smRPi1LTR is a newly evolved small RNA, resulting due to rapid rearrangement of LTR and is an intermediate small RNA species transitioning from siRNAs to microRNAs (miRNAs). The Pi-responsive small RNAs and their target genes are possibly involved in the development or regulation of adaptive response to phosphate starvation (Hsieh et al., 2009). The extent of pervasiveness of TE-derived siRNA in host gene expression is demonstrated by the *Athila6*-derived siRNA854 which shares partial complementarity with the target gene *UPB1b*. The latter is speculated to function in the translational inhibition of TE activity under stress, and *Athila6* might have retained the siRNA854 sequence as a mechanism to silence *UPB1b* instead (Hsieh et al., 2009). The same study estimated the total number of genes targeted by TE derived siRNAs to lie between 20 and 300. In rice, Small RNA siR441 and siR446 are derived from the MITE Stowaway-1 through OsDCL3a. These exhibit a high degree of similarity to a 21 nucleotide sequence present in the 3′ UTR of target stress responsive genes including MAIF1 (Yan et al., 2010).

In maize, critical insights into the role of TEs as determinants of natural variation for stress tolerance, exists in form of a MITE inserted in the promoter of a NAC gene ZmNAC111 (Mao et al., 2015). This example is particularly interesting because this 82-bp MITE insertion in the promoter down regulates its expression, not only though the insertional disruption, but also by acting as the source of 21–24 nt siRNA, which lead to silencing of this locus by the RdDM pathway & H3K9 dimethylation. The ZmNAC111 overexpression maize lines exhibit enhanced drought tolerance at the seedling stage, improves water-use efficiency and upregulation of drought-responsive genes under water stress (Mao et al., 2015). In sugarcane, differential mapping patterns was observed for sRNA and mRNA which showed peaks of RNA mapping in a region downstream to an LTR retrotransposon. Promoters within the 3′ LTR region may be driving expression of this region in an allelic dependent manner (de Setta et al., 2014). Thus TEs, acting through small non-coding RNA can provide a widespread regulatory influence over host gene expression pertaining to stress tolerance.

Despite the predominance of LTR retroelements and MITEs in stress regulation, non-LTRs are not completely far behind. For example, in the resurrection plant *Craterostigma plantagineum* activation tagging identified *Craterostigma* desiccation tolerant (CDT-1), a dehydration-related ABA-inducible gene which confer desiccation tolerance without ABA pre-treatment of the callus (Furini et al., 1997). Related to a family of dehydration responsive retroelements, CDT-1 represents an evolutionary novelty; it is an intron-less multi-copy gene flanked by direct repeats and possesses a very small ORF which is not translated into a protein; instead it encodes a double-stranded 21-bp short interfering RNA (siRNA), which regulate the expression of ABA- and dehydration-responsive genes leading to desiccation tolerance (Hilbricht et al., 2008). Interestingly, the default expression of CDT-1 is only under dehydration stress in vegetative tissues, and upon ABA treatment in the callus. CDT-1 thus serves to illustrate the critical link between environment and genome; CDT-1 is transcribed and transposed under dehydration, and the increasing numbers of such insertions into a stress responsive transcribed locus will eventually lead to a progressively higher level of CDT-1 siRNA, ultimately triggering desiccation tolerance (Hilbricht et al., 2008). This example is particularly important as it demonstrates how TEs are critical in plant evolution and stress response-not only TEs drive stress induced genome dynamics, but also function as the material for evolutionary innovation directed toward plant stress tolerance.

### Recruitment of TE coding sequences as host stress response genes

While many examples involving TE protein exaptation exist in mammalian and other vertebrate systems (Volff, 2006; Hoen and Bureau, 2012), those implicated in plant stress response are still scarce. One such example is provided by the Rim2 gene, which is expressed in response to fungal elicitors, implying a role in plant defense (He et al., 2000). Interestingly, the Rim2 protein exhibits considerable sequence homology to the TNP2 transposase of the CACTA TE family. It is present in at least four copies in the modern rice varieties, suggesting that the progenitor element was mobile at some time point prior to becoming inactive (He et al., 2000). Similarly, the *Arabidopsis* AtCopeg1 (Copia evolved gene 1), has evolved from AtCopia95_I, and encodes AtCopia95 polyprotein (Duan et al., 2008). Initially identified as a salt inducible transcript, AtCopeg1 is an intron containing gene, having two alternative 3′ ends with specific expression in leaves and roots. Further, it shows strong fluctuations in response to phosphate (Pi) starvation, nitrate, potassium, or iron starvation. Moreover, external treatment with hormones and hormone analogs, was also discovered to bring about marked changes in its expression, suggesting a possible involvement in the cross talk between various hormone and nutrient stress responsive pathway (Duan et al., 2008).

Despite the existing wealth of information regarding upregulation of TEs under various stresses and environmental challenges, a cause and effect relationship between TE activation and stress adaptation is yet to be established. The aforementioned examples have provided an insight into the possible mechanisms of TE-mediated control of host stress response machinery (Figure 2). Viewed together, it seems likely that the relationship between TE activation and stress response pathways is an intricately complex one, and it will take extensive evidence to prove TEs as McClintock's de facto controlling elements.

### From parasitic to phenotypic: toward an applied front for TEs

The abovementioned studies highlight TEs as the yet untapped reservoirs of genetic variation and phenotypic diversity. The question that arises now is how can TEs be exploited for crop improvement and increasing crop productivity. An interesting and relatively less explored theme is the selectivity in transposition. The rice mPing, an active TE in rice, was shown to be exclusively in single copy regions (Jiang et al., 2003). Furthermore, the comprehensive sequence analysis of, 1664 mPing insertion sites in rice genome and their impact on the expression profile of 710 genes revealed a surprising avoidance of exon insertions and a preference for insertion into 5′ flanking regions instead. The lack of deleterious effects associated with such a TE burst demonstrates how TEs can establish stress-responsive networks (Naito et al., 2009). Tf1, an LTR retrotransposon in *Schizosaccharomyces pombe*, exhibits a propensity for integration into promoters of stress response genes (Feng et al., 2012). Moreover, only the host genes subject to heat induction themselves, could be activated by Tf1 integration, suggesting a synergy of Tf1 enhancer sequence with the stress response elements of target promoters (Feng et al., 2012). While such a selectivity component in TE activity is in agreement with the proposed regulatory role, the area is still nascent and would require further investigation. A recent review suggested the development of controlled transposition for crop improvement (Paszkowski, 2015). The stress-induced, TE mediated distribution of regulatory elements, can potentially create and fine-tune many regulatory networks responsive to the original stress. More importantly however, the dynamic and possibly heritable restructuring of the host epigenetic landscape can create stress-inducible epiallelic switches. Based on this information, can the deployment of an inducible TE create selective changes to stress responsive QTLs in stress-susceptible cultivars, thus rewiring the transcriptional circuits for tolerance? Can the controlled transposition system be used for creating epigenetic diversity capable of conferring stress tolerance? Such inducible diversity can theoretically be paving the path for phenotypic plasticity—the ability to adjust to seasonal variations so as to maintain homeostasis during development (Rathcke and Lacey, 1985; Paszkowski, 2015). The latter is crucial for the successful adaptation and survival of crop cultivars facing stress. Therefore, controlled transposition in crop plants may potentially generate cultivars with novel variation in response to a particular stress, with implications for sustainable agriculture (Bloomfield et al., 2014).

A second area where studies of TE can directly assist the crop improvement programs is through molecular marker approach. This is especially significant in the case of polyploidy crops with frequent stress-induced genomic rearrangements. The presence of TEs, often close to or within the stress responsive QTLs, especially plant defense genes, along with the traditional attributes of a molecular marker, makes them the markers of choice for diversity studies and trait mapping (Kalendar et al., 2011; Alzohairy et al., 2014). The mapping of “spikelet-tipped bristles” (*stb*), which is involved in determining grain number per panicle in foxtail millet, *Setaria italica* (L.) P. Beauv represents one such example (Sato et al., 2013). Using a combination of transposon display and SSR markers in the F2 population of a cross between a Taiwanese and Japanese landrace, stb1 was successfully mapped to chromosome 2 (Sato et al., 2013). In recognition of their utility in mapping studies and diversity analysis, certain TE based markers, such as IRAP (Inter Retrotransposon Amplification Polymorphism), REMAP (Retrotransposon-microsatellite amplified polymorphism) and SSAP (Sequence Specific Amplification Polymorphism) have been used to some extent in crop improvement programs (Table 2, Manninen et al., 2000; Queen et al., 2004). A more high-throughput application of TE based polymorphism in diversity analysis was shown in soybean for GmCHX1, previously referred to as GmSALT3 (Guan et al., 2014). The same locus was further probed through whole-genome resequencing (WGRS) on 106 diverse soybean lines and three major structural variants and allelic variation were identified (Patil et al., 2016). These SNPs were then utilized for the design of six KASPar (Kompetitive allele-specific polymerase chain reaction) assays. The haplotype analysis of 104 soybean lines revealed a strong correlation between the genotype and salinity associated phenotype. The high resolution (>91%) and polymorphism (>98%) offered by these markers demonstrate how TE-generated polymorphism can be utilized in crop improvement programs.

**Table 2:** **Selected examples demonstrating the application of transposon markers in crop improvement for disease resistance**.

**Marker technique**	**Transposon element**	**Plant species**	**Study**	**References**
SSAP	Tao1, Tao2	*Anacardium occidentale* L.	Assessment of polymorphism in seregating F1 progeny of genetic cross for nut size, resistance to anthracnose and black mould	Syed et al., 2005
IRAP and REMAP	Bagy2, Wilma, Sumana, Sabrina, Haight	*Triticum dicoccoides*	MAS (Marker assisted selection) for stripe rust resistance	Mandoulakani et al., 2015
IRAP and REMAP	Reme1	*Cucumis melo* L.	Development of markers associated with the melon populations resistant to ZYMV and potential use for introgression in MAS	Mandoulakani and Bernousi, 2015
IRAP and REMAP	LTR 6150 d Nikita Carica papaya	*Carica papaya*	Identification of papaya breeding material resistance to PRSV and potential application for use in Molecular Assisted Breeding (MAB) and MAS	Rashid et al., 2014

The generation of genetic variability in crops with a narrow genetic base, or targeting traits that are not normally present in the crop germplasm in question, can be accomplished by the use of irradiation technology (Tanaka, 1999). Exposure to irradiation generates mutations on a global scale, which constitutes a genomic shock-like situation (McClintock, 1984). Under such conditions, TEs are mobilized globally creating widespread changes in the host genome, some of these changes might be beneficial and selected through evolutionary sieve to affect heritable changes in the host genome (McClintock, 1984; Ito et al., 2011; Fedoroff, 2012). This rationale serves as the basis of *in vitro* mutagenesis system for crop improvement, where such beneficial induced mutations are selected and maintained through multiple cycles, a prominent example being the low-cadmium rice (Ishikawa et al., 2012). This system holds special significance for polyploid crops, for which breeding is difficult, such as sugarcane. We have successfully applied the *in vitro* mutagenesis system to obtain high sugar- (Mirajkar et al., 2016) as well as salt-tolerant mutants (Nikam et al., 2015). Taking into view that TE mobilization is one of the major events under radiation exposure, further efforts toward can be directed toward enhancing the transposon mobility and transposition efficiency so as to generate heritable mutations with a definite, selectable phenotype.

## Conclusions

Long viewed as “parasites” hazardous to host survival, TEs have since been recognized for their role in plant evolution and stress responses (Bui and Grandbastien, 2012; Fedoroff, 2012; Lisch, 2012). Multiple studies spanning various plant systems, in particular, ONSEN, and mPing, suggest that TEs not only rewire host transcriptional circuits in times of stress, but the extensive genomic rearrangements mediated by such TE bursts shapes genome architecture, ultimately leading to speciation and evolution of plant genomes (McClintock, 1950; Fedoroff, 2012; Lisch, 2012; Bonchev and Parisod, 2013). The host epigenetic processes are thought to have evolved to counter and repress their activity, highlighting their still prevalent image as deleterious to host. However, it is now recognized that under stress, the relaxation of such epigenetic constrains permits TEs to perpetuate their stress-inducible regulatory sequences to hostgenes. TEs also spread their epigenetic signatures to the surrounding new loci, and combined with the generation of regulatory small RNA contribute to epigenetic regulation of host genes as well. As seen in case of ONSEN and mPing, the stress induced transcription and transposition of TEs epigenetic feedback mechanism is at play that re-establishes methylation and silencing of TEs following recovery from stress. This permits a truly dynamic reshaping of the host epigenetic landscape under prevailing environmental stimulus. The recognition of the underlying active regulatory potential of TEs could pave the way for their successful application in crop improvement programs in multiple ways. Clearly, the connection between TEs and host stress response is an exceedingly complex and multifaceted one, and needs further exploration for their potential to be fully realized.

## Perspectives

The ever-growing world population places a proportionately large demand on agriculture for subsistence. Maintaining the crop production is proving to be a hard task in the face of changing climatic scene. The concept of genetic diversity and phenotypic plasticity thus becomes crucial, in the context of developing crops resilient to such climate changes, as the lack of adaptive plasticity can drive a particular cultivar to decline. From a molecular perspective, this phenotypic plasticity is conferred by epigenetic processes, speculated to have evolved to counter viruses and TEs. The latter are known to contribute to the transcriptional and epigenetic landscape of their immediate neighboring regions, and are emerging as active regulators of host stress response. In times of changing environments, the rapid phenotypic plasticity conferred by host epigenetic responses may translate in stress-adaptation. Controlled transposition-the deployment of inducible TE to specific host loci for rewiring transcriptional and epigenetic network scan therefore lead to generation of novel genetic variation and phenotypic plasticity aimed toward stress tolerance. The usefulness of TEs for mapping studies and mutation breeding only adds to their applicability in crop improvement programs. It is being realized that investigation of TE regulation of host transcriptional networks and epigenetic responses is critical for traversing the translational bridge from a purely basic study of TEs to the applied field of stress adaptation and crop productivity.

## Author contributions

PN took the lead in writing with inputs from AR during the writing process. PS coordinated the entire work and finalization of manuscript.

## Funding

Departmental funding from BARC.

### Conflict of interest statement

The authors declare that the research was conducted in the absence of any commercial or financial relationships that could be construed as a potential conflict of interest.

## References

[B1] Akhunov E. Akhunova A. Dvorak J. (2007). Mechanisms and rates of birth and death of dispersed duplicated genes during the evolution of a multigene family in diploid and tetraploid wheats. Mol. Biol. Evol. 24, 539–550. 10.1093/molbev/msl183 17135334

[B2] Alzohairy A. Gyulai G. Ramadan M. F. Edris S. Sabir J. S. Jansen R. K. (2014). Retrotransposon-based molecular markers for assessment of genomic diversity. Funct. Plant Biol. 41, 781 10.1071/FP13351 32481032

[B3] Anca I. Fromentin J. Bui Q. T. Mhiri C. Grandbastien M. A. Simon-Plas F.. (2014). Different tobacco retrotransposons are specifically modulated by the elicitor cryptogein and reactive oxygen species. J. Plant Physiol. 171, 1533–1540. 10.1016/j.jplph.2014.07.003 25128785

[B4] Ansari K. Walter S. Brennan J. M. Lemmens M. Kessans S. McGahern A.. (2007). Retrotransposon and gene activation in wheat in response to mycotoxigenic and non-mycotoxigenic-associated *Fusarium* stress. Theor. Appl. Genet. 114, 927–937. 10.1007/s00122-006-0490-0 17256175

[B5] Arabidopsis Genome Initiative (2000). Analysis of the genome sequence of the flowering plant *Arabidopsis thaliana*. Nature 408, 796–815. 10.1038/35048692 11130711

[B6] Avramova Z. (2015). Transcriptional ‘memory’ of a stress: transient chromatin and memory (epigenetic) marks at stress-response genes. Plant J. 83, 149–159. 10.1111/tpj.12832 25788029

[B7] Ay N. Clauss K. Barth O. Humbeck K. (2008). Identification and characterization of novel senescence-associated genes from barley (*Hordeum vulgare*) primary leaves. Plant Biol. 10, 121–135. 10.1111/j.1438-8677.2008.00092.x 18721317

[B8] Barbaglia A. Klusman K. M. Higgins J. Shaw J. R. Hannah L. C. Lal S. K. (2012). Gene capture by helitron transposons reshuffles the transcriptome of maize. Genetics 190, 965–975. 10.1534/genetics.111.136176 22174072 PMC3296258

[B9] Barret P. Brinkman M. Beckert M. (2006). A sequence related to rice Pong transposable element displays transcriptional activation by *in vitro* culture and reveals somaclonal variations in maize. Genome 49, 1399–1407. 10.1139/g06-109 17426755

[B10] Ben-David S. Yaakov B. Kashkush K. (2013). Genome-wide analysis of short interspersed nuclear elements SINES revealed high sequence conservation, gene association and retrotranspositional activity in wheat. Plant J. 76, 201–210. 10.1111/tpj.12285 23855320 PMC4223381

[B11] Berg J. Appiano M. Martínez M. S. Hermans F. W. Vriezen W. H. Visser R. G.. (2015). A transposable element insertion in the susceptibility gene CsaMLO8 results in hypocotyl resistance to powdery mildew in cucumber. BMC Plant Biol. 15:243. 10.1186/s12870-015-0635-x 26453551 PMC4600303

[B12] Bhattacharyya M. K. Smith A. M. Ellis T. H. Hedley C. Martin C. (1990). The wrinkled-seed character of pea described by Mendel is caused by a transposon-like insertion in a gene encoding starch-branching enzyme. Cell 60, 115–122. 10.1016/0092-8674(90)90721-P 2153053

[B13] Bloomfield J. A. Rose T. J. King G. J. (2014). Sustainable harvest: managing plasticity for resilient crops. Plant Biotechnol. J. 12, 517–533. 10.1111/pbi.12198 24891039 PMC4207195

[B14] Bolger A. Scossa F. Bolger M. E. Lanz C. Maumus F. Tohge T.. (2014). The genome of the stress-tolerant wild tomato species *Solanum pennellii*. Nat. Genet. 46, 1034–1038. 10.1038/ng.3046 25064008 PMC7036041

[B15] Bonchev G. Parisod C. (2013). Transposable elements and microevolutionary changes in natural populations. Mol. Ecol. Resour. 13, 765–775. 10.1111/1755-0998.12133 23795753

[B16] Bouvet G. Jacobi V. Plourde K. Bernier L. (2008). Stress-induced mobility of OPHIO1 and OPHIO2, DNA transposons of the Dutch elm disease fungi. Fungal Genet. Biol. 45, 565–578. 10.1016/j.fgb.2007.12.007 18255325

[B17] Bruce T. Matthes M. Napier J. Pickett J. (2007). Stressful “memories” of plants: evidence and possible mechanisms. Plant Sci. 173, 603–608. 10.1016/j.plantsci.2007.09.002

[B18] Buchmann R. Asad S. Wolf J. N. Mohannath G. Bisaro D. M. (2009). Geminivirus AL2 and L2 proteins suppress transcriptional gene silencing and cause genome-wide reductions in cytosine methylation. J. Virol. 83, 5005–5013. 10.1128/JVI.01771-08 19279102 PMC2682068

[B19] Bui Q. T. Grandbastien M.-A. (2012). LTR retrotransposons as controlling elements of genome response to stress?, in Plant Transposable Elements: Impact on Genome Structure and Function Topics in Current Genetics, eds Grandbastien M.-A. Casacuberta J. M. (Berlin; Heidelberg: Springer), 273–296.

[B20] Bundock P. Hooykaas P. (2005). An Arabidopsis hAT-like transposase is essential for plant development. Nature 436, 282–284. 10.1038/nature03667 16015335

[B21] Butelli E. Licciardello C. Zhang Y. Liu J. Mackay S. Bailey P.. (2012). Retrotransposons control fruit-specific, cold-dependent accumulation of anthocyanins in blood oranges. Plant Cell 24, 1242–1255. 10.1105/tpc.111.095232 22427337 PMC3336134

[B22] Casacuberta J. Santiago N. (2003). Plant LTR-retrotransposons and MITEs: control of transposition and impact on the evolution of plant genes and genomes. Gene 311, 1–11. 10.1016/S0378-1119(03)00557-2 12853133

[B23] Castrillo G. Sánchez-Bermejo E. de Lorenzo L. Crevillén P. Fraile-Escanciano A. Mohan T. C.. (2013). WRKY6 transcription factor restricts arsenate uptake and transposon activation in *Arabidopsis*. Plant Cell 25, 2944–2957. 10.1105/tpc.113.114009 23922208 PMC3784590

[B24] Cavrak V. V. Lettner N. Jamge S. Kosarewicz A. Bayer L. M. Scheid O. M. (2014). How a retrotransposon exploits the plant's heat stress response for its activation. PLoS Genet. 10:e1004115. 10.1371/journal.pgen.1004115 24497839 PMC3907296

[B25] Cowan R. Hoen D. Schoen D. Bureau T. (2005). MUSTANG is a novel family of domesticated transposase genes found in diverse angiosperms. Mol. Biol. Evol. 22, 2084–2089. 10.1093/molbev/msi202 15987878

[B26] de Araujo P. Rossi M. Jesus E. M. Saccaro N. L. Kajihara D. Massa R.. (2005). Transcriptionally active transposable elements in recent hybrid sugarcane. Plant J. 44, 707–717. 10.1111/j.1365-313X.2005.02579.x 16297064

[B27] de Jesus E. M. Cruz E. A. Cruz G. M. Van Sluys M. A. (2012). Diversification of hAT transposase paralogues in the sugarcane genome. Mol. Genet. Genomics 287, 205–219. 10.1007/s00438-011-0670-8 22228195 PMC3285750

[B28] de Setta N. Monteiro-Vitorello C. B. Metcalfe C. J. Cruz G. M. Del Bem L. E. Vicentini R.. (2014). Building the sugarcane genome for biotechnology and identifying evolutionary trends. BMC Genomics 15:540. 10.1186/1471-2164-15-540 24984568 PMC4122759

[B29] Dong H. Zhang L. Zheng K. L. Yao H. G. Chen J. Yu F. C.. (2012). A Gaijin-like miniature inverted repeat transposable element is mobilized in rice during cell differentiation. BMC Genomics 13:135. 10.1186/1471-2164-13-135 22500940 PMC3352178

[B30] Dong Y. Lu X. Song W. Shi L. Zhang M. Zhao H.. (2011). Structural characterization of helitrons and their stepwise capturing of gene fragments in the maize genome. BMC Genomics 12:1. 10.1186/1471-2164-12-609 22177531 PMC3288121

[B31] Doolittle W. F. Sapienza C. (1980). Selfish genes, the phenotype paradigm and genome evolution. Nature 284, 601–603. 10.1038/284601a0 6245369

[B32] Du C. Fefelova N. Caronna J. He L. Dooner H. K. (2009). The polychromatic Helitron landscape of the maize genome. Proc. Natl Acad. Sci. U.S.A. 106, 19916–19921. 10.1073/pnas.0904742106 19926866 PMC2785267

[B33] Duan K. Ding X. Zhang Q. Zhu H. Pan A. Huang J. (2008). AtCopeg1, the unique gene originated from AtCopia95 retrotransposon family, is sensitive to external hormones and abiotic stresses. Plant Cell Rep. 27, 1065–1073. 10.1007/s00299-008-0520-2 18309491

[B34] Eaton C. J. Cox M. P. Ambrose B. Becker M. Hesse U. Schardl C. L.. (2010). Disruption of signaling in a fungal-grass symbiosis leads to pathogenesis. Plant Physiol. 153, 1780–1794. 10.1104/pp.110.158451 20519633 PMC2923905

[B35] El Baidouri M. Carpentier M. C. Cooke R. Gao D. Lasserre E. Llauro C.. (2014). Widespread and frequent horizontal transfers of transposable elements in plants. Genome Res. 24, 831–838. 10.1101/gr.164400.113 24518071 PMC4009612

[B36] Fedoroff N. V. (2012). Transposable elements, epigenetics, and genome evolution. Science 338, 758–767. 10.1126/science.338.6108.758 23145453

[B37] Felice B. Wilson R. Argenziano C. Kafantaris I. Conicella C. (2009). A transcriptionally active copia-like retroelement in *Citrus limon*. Cell. Mol. Biol. Lett. 14, 289–304. 10.2478/s11658-008-0050-5 19115051 PMC6275675

[B38] Feng G. Leem Y. Levin H. (2012). Transposon integration enhances expression of stress response genes. Nucleic Acids Res. 41, 775–789. 10.1093/nar/gks1185 23193295 PMC3553992

[B39] Feschotte C. (2008). Transposable elements and the evolution of regulatory networks. Nat. Rev. Genet. 9, 397–405. 10.1038/nrg2337 18368054 PMC2596197

[B40] Finatto T. De Oliveira A. C. Chaparro C. Da Maia L. C. Farias D. R. Woyann L. G.. (2015). Abiotic stress and genome dynamics: specific genes and transposable elements response to iron excess in rice. Rice 8, 1. 10.1186/s12284-015-0045-6 25844118 PMC4385019

[B41] Finnegan D. (1989). Eukaryotic transposable elements and genome evolution. Trends Genet. 5, 103–107. 10.1016/0168-9525(89)90039-5 2543105

[B42] Fukai E. Dobrowolska A. D. Madsen L. H. Madsen E. B. Umehara Y. Kouchi H.. (2008). Transposition of a 600 thousand-year-old LTR retrotransposon in the model legume *Lotus japonicus*. Plant Mol. Biol. 68, 653–663. 10.1007/s11103-008-9397-2 18802778

[B43] Fukai E. Umehara Y. Sato S. Endo M. Kouchi H. Hayashi M.. (2010). Derepression of the plant Chromovirus LORE1 induces germline transposition in regenerated plants. PLoS Genet. 6:e1000868. 10.1371/journal.pgen.1000868 20221264 PMC2832683

[B44] Furini A. Koncz C. Salamini F. Bartels D. (1997). High level transcription of a member of a repeated gene family confers dehydration tolerance to callus tissue of *Craterostigma plantagineum*. EMBO J. 16, 3599–3608. 10.1093/emboj/16.12.3599 9218801 PMC1169984

[B45] Gladyshev E. A. Arkhipova I. R. (2007). Telomere-associated endonuclease-deficient *Penelope*-like retroelements in diverse eukaryotes. Proc. Natl. Acad. Sci. U.S.A. 104, 9352–9357. 10.1073/pnas.0702741104 17483479 PMC1890498

[B46] Grandbastien M. A. Audeon C. Bonnivard E. Casacuberta J. M. Chalhoub B. Costa A. P.. (2005). Stress activation and genomic impact of Tnt1 retrotransposons in Solanaceae. Cytogenet. Genome Res. 110, 229–241. 10.1159/000084957 16093677

[B47] Grandbastien M. A. Lucas H. Morel J. B. Mhiri C. Vernhettes S. Casacuberta J. M. (1997). The expression of the tobacco Tnt1 retrotransposon is linked to plant defense responses Genetica 100, 241–252. 9440277

[B48] Guan R. Qu Y. Guo Y. Yu L. Liu Y. Jiang J.. (2014). Salinity tolerance in soybean is modulated by natural variation in GmSALT3. Plant J. 80, 937–950. 10.1111/tpj.12695 25292417

[B49] Guimarγes P. Brasileiro A. C. Morgante C. V. Martins A. C. Pappas G. Silva O. B.. (2012). Global transcriptome analysis of two wild relatives of peanut under drought and fungi infection. BMC Genomics 13:387. 10.1186/1471-2164-13-387 22888963 PMC3496627

[B50] Gupta S. Gallavotti A. Stryker G. A. Schmidt R. J. Lal S. K. (2005). A novel class of Helitron-related transposable elements in maize contain portions of multiple pseudogenes. Plant Mol. Biol. 57, 115–127. 10.1007/s11103-004-6636-z 15821872

[B51] Hayashi K. Yoshida H. (2009). Refunctionalization of the ancient rice blast disease resistance gene Pit by the recruitment of a retrotransposon as a promoter. Plant J. 57, 413–425. 10.1111/j.1365-313X.2008.03694.x 18808453

[B52] He L. Dooner H. (2009). Haplotype structure strongly affects recombination in a maize genetic interval polymorphic for Helitron and retrotransposon insertions. Proc. Natl. Acad. Sci. 106, 8410–8416. 10.1073/pnas.0902972106 19416860 PMC2688972

[B53] He P. Ma Y. Zhao G. Dai H. Li H. Chang L.. (2009). FaRE1: a transcriptionally active Ty1-copia retrotransposon in strawberry. J. Plant Res. 123, 707–714. 10.1007/s10265-009-0290-0 20020171

[B54] He Z. Dong H. T. Dong J. X. Li D. B. Ronald P. C. (2000). The rice Rim2 transcript accumulates in response to *Magnaporthe grisea* and its predicted protein product shares similarity with TNP2-like proteins encoded by CACTA transposons. Mol. Gen. Genet. 264, 2–10. 10.1007/s004380000278 11016827

[B55] Hilbricht T. Varotto S. Sgaramella V. Bartels D. Salamini F. Furini A. (2008). Retrotransposons and siRNA have a role in the evolution of desiccation tolerance leading to resurrection of the plant *Craterostigma plantagineum*. New Phytol. 179, 877–887. 10.1111/j.1469-8137.2008.02480.x 18482228

[B56] Hirayama T. Shinozaki K. (2010). Research on plant abiotic stress responses in the post-genome era: past, present and future. Plant J. 61, 1041–1052. 10.1111/j.1365-313X.2010.04124.x 20409277

[B57] Hirochika H. (1993). Activation of tobacco retrotransposons during tissue culture. EMBO J. 12, 2521. 8389699 10.1002/j.1460-2075.1993.tb05907.x PMC413490

[B58] Hirochika H. Sugimoto K. Otsuki Y. Tsugawa H. Kanda M. (1996). Retrotransposons of rice involved in mutations induced by tissue culture. Proc. Natl Acad. Sci. U.S.A. 93, 7783–7788. 8755553 10.1073/pnas.93.15.7783 PMC38825

[B59] Hoen D. R. Bureau T. E. (2012). Transposable element exaptation in plants, in Plant Transposable Elements: Impact on Genome Structure and Function, eds Grandbastien M.-A. Casacuberta J. M. (Berlin; Heidelberg: Springer), 219–251.

[B60] Hsieh L. Lin S. I. Shih A. C. Chen J. W. Lin W. Y. Tseng C. Y.. (2009). Uncovering small RNA-mediated responses to phosphate deficiency in *Arabidopsis* by deep sequencing. Plant Physiol. 151, 2120–2132. 10.1104/pp.109.147280 19854858 PMC2785986

[B61] Hu T. He S. Yang G. Zeng H. Wang G. Chen Z.. (2011). Isolation and characterization of a rice glutathione S-transferase gene promoter regulated by herbicides and hormones. Plant Cell Rep. 30, 539–549. 10.1007/s00299-010-0964-z 21153026

[B62] Humphry M. Reinstaedler A. Ivanov S. Bisseling T. O. Panstruga R. (2011). Durable broad-spectrum powdery mildew resistance in pea er1 plants is conferred by natural loss-of-function mutations in PsMLO1. Mol. Plant Pathol. 12, 866–878. 10.1111/j.1364-3703.2011.00718.x 21726385 PMC6640514

[B63] International Barley Genome Sequencing Consortium (2012). A physical, genetic and functional sequence assembly of the barley genome. Nature 491, 711–716. 10.1038/nature11543 23075845

[B64] International Rice Genome Consortium (2005). The map-based sequence of the rice genome. Nature 436, 793. 10.1038/nature03895 16100779

[B65] Ishiguro S. Ogasawara K. Fujino K. Sato Y. Kishima Y. (2014). Low temperature-responsive changes in the anther transcriptome's repeat sequences are indicative of stress sensitivity and pollen sterility in rice strains. Plant Physiol. 164, 671–682. 10.1104/pp.113.230656 24376281 PMC3912097

[B66] Ishikawa S. Ishimaru Y. Igura M. Kuramata M. Abe T. Senoura T.. (2012). Ion-beam irradiation, gene identification, and marker-assisted breeding in the development of low-cadmium rice. Proc. Natl Acad. Sci. U.S.A. 109, 19166–19171. 10.1073/pnas.1211132109 23132948 PMC3511095

[B67] Ito H. Gaubert H. Bucher E. Mirouze M. Vaillant I. Paszkowski J. (2011). An siRNA pathway prevents transgenerational retrotransposition in plants subjected to stress. Nature 472, 115–119. 10.1038/nature09861 21399627

[B68] Jeung J. U. Cho S. K. Lee S. J. Shin J. S. (2005). Characterization of Ty3-gypsy-like elements in *Hibiscus syriacus*. Molecules Cells 19, 318–327. 15995347

[B69] Jiang N. Bao Z. Temnykh S. Cheng Z. Jiang J. Wing R. A.. (2002). Dasheng: a recently amplified nonautonomous long terminal repeat element that is a major component of pericentromeric regions in rice. Genetics 161, 1293–1305. 12136031 10.1093/genetics/161.3.1293 PMC1462185

[B70] Jiang N. Bao Z. Zhang X. Eddy S. R. Wessler S. R. (2004). Pack-MULE transposable elements mediate gene evolution in plants. Nature 431, 569–573. 10.1038/nature02953 15457261

[B71] Jiang N. Bao Z. Zhang X. Hirochika H. Eddy S. R. McCouch S. R.. (2003). An active DNA transposon family in rice. Nature 421, 163–167. 10.1038/nature01214 12520302

[B72] Jiang N. Wessler S. (2001). Insertion preference of maize and rice miniature inverted repeat transposable elements as revealed by the analysis of nested elements. Plant Cell 13:2553. 10.1105/tpc.13.11.2553 11701888 PMC139471

[B73] Jiao Y. Deng X. (2007). A genome-wide transcriptional activity survey of rice transposable element-related genes. Genome Biol. 8:R28. 10.1186/gb-2007-8-2-r28 17326825 PMC1852403

[B74] Johns M. A. Mottinger J. Freeling M. (1985). A low copy number, copia-like transposon in maize. EMBO J. 4, 1093. 2988938 10.1002/j.1460-2075.1985.tb03745.x PMC554309

[B75] Joly-Lopez Z. Forczek E. Hoen D. R. Juretic N. Bureau T. E. (2012). A gene family derived from transposable elements during early angiosperm evolution has reproductive fitness benefits in *Arabidopsis thaliana*. PLoS Genet. 8:e1002931. 10.1371/journal.pgen.1002931 22969437 PMC3435246

[B76] Juretic N. Hoen D. R. Huynh M. L. Harrison P. M. Bureau T. E. (2005). The evolutionary fate of MULE-mediated duplications of host gene fragments in rice. Genome Res. 15, 1292–1297. 10.1101/gr.4064205 16140995 PMC1199544

[B77] Kalendar R. Flavell A. J. Ellis T. H. Sjakste T. Moisy C. Schulman A. H. (2011). Analysis of plant diversity with retrotransposon-based molecular markers. Heredity 106, 520–530. 10.1038/hdy.2010.93 20683483 PMC3183911

[B78] Kalendar R. Tanskanen J. Immonen S. Nevo E. Schulman A. H. (2000). Genome evolution of wild barley (*Hordeum spontaneum*) by BARE-1 retrotransposon dynamics in response to sharp microclimatic divergence. Proc. Natl. Acad. Sci. 97, 6603–6607. 10.1073/pnas.110587497 10823912 PMC18673

[B79] Kapitonov V. V. Jurka J. (2001). Rolling-circle transposons in eukaryotes. Proc. Natl. Acad. Sci. 98, 8714–8719. 10.1073/pnas.151269298 11447285 PMC37501

[B80] Kejnovsky E. Hawkins J. Feschotte C. (2012). Plant transposable elements: biology and evolution, in Plant Genome Diversity, eds Wendel J. F. Greilhuber J. Dolezel J. Leitch I. J. (New York, NY Springer), 17–34.

[B81] Kidwell M. Lisch D. (1997). Transposable elements as sources of variation in animals and plants. Proc. Natl. Acad. Sci. 94, 7704–7711. 10.1073/pnas.94.15.7704 9223252 PMC33680

[B82] Kikuchi K. Terauchi K. Wada M. Hirano H. (2003). The plant MITE mPing is mobilized in anther culture. Nature 421, 167–170. 10.1038/nature01218 12520303

[B83] Kinoshita T. Seki M. (2014). Epigenetic memory for stress response and adaptation in plants. Plant Cell Physiol. 55, 1859–1863. 10.1093/pcp/pcu125 25298421

[B84] Kinoshita Y. Saze H. Kinoshita T. Miura A. Soppe W. J. Koornneef M.. (2007). Control of FWA gene silencing in *Arabidopsis thaliana* by SINE-related direct repeats. Plant J. 49, 38–45. 10.1111/j.1365-313X.2006.02936.x 17144899

[B85] Krom N. Recla J. Ramakrishna W. (2008). Analysis of genes associated with retrotransposons in the rice genome. Genetica 134, 297–310. 10.1007/s10709-007-9237-3 18066688

[B86] Lal S. Giroux M. J. Brendel V. Vallejos C. E. Hannah L. C. (2003). The maize genome contains a helitron insertion. Plant Cell 15, 381–391. 10.1105/tpc.008375 12566579 PMC141208

[B87] Lal S. Hannah L. (2005). Plant genomes: massive changes of the maize genome are caused by Helitrons. Heredity 95, 421–422. 10.1038/sj.hdy.6800764 16222326

[B88] Lee S. I. Kim N. S. (2014). Transposable elements and genome size variations in plants. Genomics Inform. 12, 87–97. 10.5808/GI.2014.12.3.87 25317107 PMC4196380

[B89] Leeton P. R. Smyth D. R. (1993). An abundant LINE-like element amplified in the genome of *Lilium speciosum*. Mol. Gen. Genet. 237, 97–104. 10.1007/bf00282789 7681139

[B90] Li J. Wang Z. Peng H. Liu Z. (2014). A MITE insertion into the 3′-UTR regulates the transcription of *TaHSP16.9* in common wheat. Crop J. 2, 381–387. 10.1016/j.cj.2014.07.001

[B91] Lim C. J. Yang K. A. Hong J. K. Choi J. S. Yun D. J. Hong J. C.. (2006). Gene expression profiles during heat acclimation in *Arabidopsis thaliana* suspension-culture cells. J. Plant Res. 119, 373–383. 10.1007/s10265-006-0285-z 16807682

[B92] Lin R. Ding L. Casola C. Ripoll D. R. Feschotte C. Wang H. (2007). Transposase-derived transcription factors regulate light signaling in Arabidopsis. Science 318, 1302–1305. 10.1126/science.1146281 18033885 PMC2151751

[B93] Lin X. Faridi N. Casola C. (2016). An ancient trans-kingdom horizontal transfer of *Penelope*-like retroelements from arthropods to conifers. Genome Biol. Evol. 8, 1252–1266. 10.1093/gbe/evw076 27190138 PMC4860704

[B94] Lin X. Long L. Shan X. Zhang S. Shen S. Liu B. (2006). In planta mobilization of mPing and its putative autonomous element Pong in rice by hydrostatic pressurization. J. Exp. Bot. 57, 2313–2323. 10.1093/jxb/erj203 16818484

[B95] Lisch D. (2012). How important are transposons for plant evolution? Nat. Rev. Genet. 14, 49–61. 10.1038/nrg3374 23247435

[B96] Liu J. He Y. Amasino R. Chen X. (2004). siRNAs targeting an intronic transposon in the regulation of natural flowering behavior in Arabidopsis. Genes Dev. 18, 2873–2878. 10.1101/gad.1217304 15545622 PMC534648

[B97] Long L. Ou X. Liu J. Lin X. Sheng L. Liu B. (2009). The spaceflight environment can induce transpositional activation of multiple endogenous transposable elements in a genotype-dependent manner in rice. J. Plant Physiol. 166, 2035–2045. 10.1016/j.jplph.2009.06.007 19628300

[B98] Lopes F. Jjingo D. da Silva C. R. Andrade A. C. Marraccini P. Teixeira J. B.. (2013). Transcriptional activity, chromosomal distribution and expression effects of transposable elements in *Coffea* genomes. PLoS ONE 8:e78931. 10.1371/journal.pone.0078931 24244387 PMC3823963

[B99] Lu C. Chen J. Zhang Y. Hu Q. Su W. Kuang H. (2012). Miniature Inverted-Repeat Transposable Elements (MITEs) have been accumulated through amplification bursts and play important roles in gene expression and species diversity in *Oryza sativa*. Mol. Biol. Evol. 29, 1005–1017. 10.1093/molbev/msr282 22096216 PMC3278479

[B100] Lu H. Dong H. T. Sun C. B. Qing D. J. Li N. Wu Z. K.. (2011). The panorama of physiological responses and gene expression of whole plant of maize inbred line YQ7-96 at the three-leaf stage under water deficit and re-watering. Theor. Appl. Genet. 123, 943–958. 10.1007/s00122-011-1638-0 21735236

[B101] Luck J. Lawrence G. J. Finnegan E. J. Jones D. A. Ellis J. G. (1998). A flax transposon identified in two spontaneous mutant alleles of theL6rust resistance gene. Plant J. 16, 365–369. 10.1046/j.1365-313x.1998.00306.x 9881156

[B102] Madsen L. Fukai E. Radutoiu S. Yost C. K. Sandal N. Schauser L.. (2005). LORE1, an active low-copy-number TY3-gypsy retrotransposon family in the model legume *Lotus japonicus*. Plant J. 44, 372–381. 10.1111/j.1365-313X.2005.02534.x 16236148

[B103] Magalhaes J. Liu J. Guimaraes C. T. Lana U. G. Alves V. M. Wang Y. H.. (2007). A gene in the multidrug and toxic compound extrusion (MATE) family confers aluminum tolerance in *Sorghum*. Nat. Genet. 39, 1156–1161. 10.1038/ng2074 17721535

[B104] Mandoulakani B. A. Bernousi I. (2015). Genetic diversity in iranian melon populations and hybrids assessed by IRAP and REMAP markers. J. Agric. Sci. Technol. 17, 1267–1277.

[B105] Mandoulakani B. A. Yaniv E. Kalendar R. Raats D. Bariana H. S. Bihamta M. R.. (2015). Development of IRAP-and REMAP-derived SCAR markers for marker-assisted selection of the stripe rust resistance gene Yr15 derived from wild emmer wheat. Theor. Appl. Genet. 128, 211–219. 10.1007/s00122-014-2422-8 25388968

[B106] Manninen O. Kalendar R. Robinson J. Schulman A. (2000). Application of BARE-1 retrotransposon markers to the mapping of a major resistance gene for net blotch in barley. Mol. Gen. Genet. 264, 325–334. 10.1007/s004380000326 11085273

[B107] Mao H. Wang H. Liu S. Li Z. Yang X. Yan J.. (2015). A transposable element in a NAC gene is associated with drought tolerance in maize seedlings. Nat. Commun. 6, 8326. 10.1038/ncomms9326 26387805 PMC4595727

[B108] Mao L. Wood T. C. Yu Y. Budiman M. A. Tomkins J. Woo S. S.. (2000). Rice transposable elements: a survey of 73,000 sequence-tagged-connectors. Genome Res. 10, 982–990. 10.1101/gr.10.7.982 10899147 PMC310901

[B109] Matsunaga W. Kobayashi A. Kato A. Ito H. (2012). The effects of heat induction and the siRNA biogenesis pathway on the transgenerational transposition of ONSEN, a copia-like retrotransposon in *Arabidopsis thaliana*. Plant Cell Physiol. 53, 824–833. 10.1093/pcp/pcr179 22173101

[B110] McClintock B. (1950). The origin and behavior of mutable loci in maize. Proc. Natl. Acad. Sci. 36, 344–355. 10.1073/pnas.36.6.344 15430309 PMC1063197

[B111] McClintock B. (1984). The significance of responses of the genome to challenge. Science 226, 792–801. 10.1126/science.15739260 15739260

[B112] Meyers B. Tingey S. V. Morgante M. (2001). Abundance, distribution, and transcriptional activity of repetitive elements in the maize genome. Genome Res. 11, 1660–1676. 10.1101/gr.188201 11591643 PMC311155

[B113] Mirajkar S. Suprasanna P. Vaidya E. (2016). Spatial distribution and dynamics of sucrose metabolising enzymes in radiation induced mutants of sugarcane. Plant Physiol. Biochem. 100, 85–93. 10.1016/j.plaphy.2015.12.018 26795733

[B114] Mirouze M. Paszkowski J. (2011). Epigenetic contribution to stress adaptation in plants. Curr. Opin. Plant Biol. 14, 267–274. 10.1016/j.pbi.2011.03.004 21450514

[B115] Morgante M. Brunner S. Pea G. Fengler K. Zuccolo A. Rafalski A. (2005). Gene duplication and exon shuffling by helitron-like transposons generate intraspecies diversity in maize. Nat. Genet. 37, 997–1002. 10.1038/ng1615 16056225

[B116] Muehlbauer G. J. Bhau B. S. Syed N. H. Heinen S. Cho S. Marshall D.. (2006). A hAT superfamily transposase recruited by the cereal grass genome. Mol. Genet. Genomics 275, 553–563. 10.1007/s00438-006-0098-8 16468023

[B117] Naito K. Cho E. Yang G. Campbell M. A. Yano K. Okumoto Y.. (2006). Dramatic amplification of a rice transposable element during recent domestication. Proc. Natl. Acad. Sci. 103, 17620–17625. 10.1073/pnas.0605421103 17101970 PMC1693796

[B118] Naito K. Zhang F. Tsukiyama T. Saito H. Hancock C. N. Richardson A. O.. (2009). Unexpected consequences of a sudden and massive transposon amplification on rice gene expression. Nature 461, 1130–1134. 10.1038/nature08479 19847266

[B119] Nakazaki T. Okumoto Y. Horibata A. Yamahira S. Teraishi M. Nishida H.. (2003). Mobilization of a transposon in the rice genome. Nature 421, 170–172. 10.1038/nature01219 12520304

[B120] Neumann P. Yan H. Jiang J. (2007). The centromeric retrotransposons of rice are transcribed and differentially processed by RNA interference. Genetics 176, 749–761. 10.1534/genetics.107.071902 17409063 PMC1894605

[B121] Ngezahayo F. Xu C. Wang H. Jiang L. Pang J. Liu B. (2009). Tissue culture-induced transpositional activity of mPing is correlated with cytosine methylation in rice. BMC Plant Biol. 9:91. 10.1186/1471-2229-9-91 19604382 PMC2715021

[B122] Nikam A. Devarumath R. Ahuja A. Babu H. Shitole M. G. Suprasanna P. (2015). Radiation-induced *in vitro* mutagenesis system for salt tolerance and other agronomic characters in sugarcane (*Saccharum officinarum* L.). Crop J. 3, 46–56. 10.1016/j.cj.2014.09.002

[B123] Ohno S. (1972). So much “junk” DNA in our genome. Brookhaven Symp. Biol. 23, 366–370. 5065367

[B124] Orgel L. E. Crick F. H. (1980). Selfish DNA: the ultimate parasite. Nature 284, 604. 10.1038/284604a0 7366731

[B125] Pan X. Li Y. Stein L. (2005). Site Preferences of insertional mutagenesis agents in *Arabidopsis*. Plant Physiol. 137, 168–175. 10.1104/pp.104.053215 15618417 PMC548848

[B126] Park K. I. Ishikawa N. Morita Y. Choi J. D. Hoshino A. Iida S. (2007). A bHLH regulatory gene in the common morning glory, Ipomoea purpurea, controls anthocyanin biosynthesis in flowers, proanthocyanidin and phytomelanin pigmentation in seeds, and seed trichome formation. Plant J. 49, 641–654. 10.1111/j.1365-313X.2006.02988.x 17270013

[B127] Paszkowski J. (2015). Controlled activation of retrotransposition for plant breeding. Curr. Opin. Biotechnol. 32, 200–206. 10.1016/j.copbio.2015.01.003 25615932

[B128] Paterson A. H. Bowers J. E. Bruggmann R. Dubchak I. Grimwood J. Gundlach H.. (2009). The sorghum bicolor genome and the diversification of grasses. Nature 457, 551–556. 10.1038/nature07723 19189423

[B129] Patil G. Do T. Vuong T. D. Valliyodan B. Lee J.-D. Chaudhary J.. (2016). Genomic-assisted haplotype analysis and the development of high-throughput SNP markers for salinity tolerance in soybean. Sci. Rep. 6:19199. 10.1038/srep19199 26781337 PMC4726057

[B130] Pecinka A. Dinh H. Baubec T. Rosa M. Lettner N. Scheid O. M. (2010). Epigenetic regulation of repetitive elements is attenuated by prolonged heat stress in *Arabidopsis*. Plant Cell 22, 3118–3129. 10.1105/tpc.110.078493 20876829 PMC2965555

[B131] Petit M. Guidat C. Daniel J. Denis E. Montoriol E. Bui Q. T.. (2010). Mobilization of retrotransposons in synthetic allotetraploid tobacco. New Phytol. 186, 135–147. 10.1111/j.1469-8137.2009.03140.x 20074093

[B132] Piégu B. Bire S. Arensburger P. Bigot Y. (2015). A survey of transposable element classification systems–a call for a fundamental update to meet the challenge of their diversity and complexity. Mol. Phylogenet. Evol. 86, 90–109. 10.1016/j.ympev.2015.03.009 25797922

[B133] Queen R. Gribbon B. James C. Jack P. Flavell A. J. (2004). Retrotransposon-based molecular markers for linkage and genetic diversity analysis in wheat. Mol. Genet. Genomics 271, 91–97. 10.1007/s00438-003-0960-x 14652738

[B134] Rakocevic A. Mondy S. Tirichine L. Cosson V. Brocard L. Iantcheva A.. (2009). MERE1, a low-copy-number copia-type retroelement in *Medicago truncatula* active during tissue culture. Plant Physiol. 151, 1250–1263. 10.1104/pp.109.138024 19656907 PMC2773106

[B135] Ramallo E. Kalendar R. Schulman A. Martínez-Izquierdo J. (2007). Reme1, a Copia retrotransposon in melon, is transcriptionally induced by UV light. Plant Mol. Biol. 66, 137–150. 10.1007/s11103-007-9258-4 18034313

[B136] Rashid K. Othman R. Y. Ali B. S. Yusof Y. M. Nezhadahmadi A. (2014). The application of irap markers in the breeding of papaya (*Carica Papaya* L.). Indian J. Sci. Technol. 7, 1720.

[B137] Rathcke B. Lacey E. P. (1985). Phenological patterns of terrestrial plants. Annu. Rev. Ecol. System. 16, 179–214. 10.1146/annurev.es.16.110185.001143

[B138] Richter T. E. Ronald P. C. (2000). The evolution of disease resistance genes. Plant Mol. Evol. 42, 195–204. 10.1007/978-94-011-4221-2_10 10688137

[B139] Rico-Cabanas L. Martínez-Izquierdo J. (2007). CIRE1, a novel transcriptionally active Ty1-copia retrotransposon from *Citrus sinensis*. Mol. Genet. Genomics 277, 365–377. 10.1007/s00438-006-0200-2 17216224

[B140] Rocheta M. Carvalho L. Viegas W. Morais-Cecílio L. (2012). Corky, a gypsy-like retrotransposon is differentially transcribed in *Quercussuber* tissues. BMC Res. Notes 5:432. 10.1186/1756-0500-5-432 22888907 PMC3465219

[B141] Sabot F. Sourdille P. Chantret N. Bernard M. (2006). Morgane, a new LTR retrotransposon group, and its subfamilies in wheats. Genetica 128, 439–447. 10.1007/s10709-006-7725-5 17028971

[B142] Sacerdot C. Mercier G. Todeschini A. Dutreix M. Springer M. Lesage P. (2005). Impact of ionizing radiation on the life cycle of *Saccharomyces cerevisiae* Ty1 retrotransposon. Yeast 22, 441–455. 10.1002/yea.1222 15849797

[B143] Salazar M. González E. Casaretto J. Casacuberta J. M. Ruiz-Lara S. (2007). The promoter of the TLC1.1 retrotransposon from *Solanum chilense* is activated by multiple stress-related signaling molecules. Plant Cell Rep. 26, 1861–1868. 10.1007/s00299-007-0375-y 17583815

[B144] Salvi S. Sponza G. Morgante M. Tomes D. Niu X. Fengler K. A.. (2007). Conserved noncoding genomic sequences associated with a flowering-time quantitative trait locus in maize. Proc. Natl. Acad. Sci. 104, 11376–11381. 10.1073/pnas.0704145104 17595297 PMC2040906

[B145] Salvi S. Tuberosa R. Chiapparino E. Maccaferri M. Veillet S. van Beuningen L.. (2002). Toward positional cloning of Vgt1, a QTL controlling the transition from the vegetative to the reproductive phase in maize. Plant Mol. Biol. 48, 601–613. 10.1023/A:1014838024509 11999837

[B146] SanMiguel P. Bennetzen J. (1998). Evidence that a recent increase in maize genome size was caused by the massive amplification of intergene retrotransposons. Ann. Bot. 82, 37–44. 10.1006/anbo.1998.0746

[B147] SanMiguel P. Tikhonov A. Jin Y. Motchoulskaia N. (1996). Nested retrotransposons in the intergenic regions of the maize genome. Science 274, 765–768. 10.1126/science.274.5288.765 8864112

[B148] Sato K. Mukainari Y. Naito K. Fukunaga K. (2013). Construction of a foxtail millet linkage map and mapping of spikelet-tipped bristles 1 (stb1) by using transposon display markers and simple sequence repeat markers with genome sequence information. Mol. Breed. 31, 675–684. 10.1007/s11032-012-9825-5

[B149] Schnable P. S. Ware D. Fulton R. S. Stein J. C. Wei F. Pasternak S.. (2009). The B73 maize genome: complexity, diversity, and dynamics. Science 326, 1112–1115. 10.1126/science.1178534 19965430

[B150] Selinger D. Chandler V. (2001). B-Bolivia, an Allele of the Maize b1 Gene with Variable Expression, Contains a High Copy Retrotransposon-Related Sequence Immediately Upstream. Plant Physiol. 125, 1363–1379. 10.1104/pp.125.3.1363 11244116 PMC65615

[B151] Shan X. Liu Z. Dong Z. Wang Y. Chen Y. Lin X.. (2005). Mobilization of the Active MITE Transposons mPing and Pong in Rice by Introgression from Wild Rice (*Zizania latifolia* Griseb.). Mol. Biol. Evol. 22, 976–990. 10.1093/molbev/msi082 15647520

[B152] Shapiro J. (2005). A 21st century view of evolution: genome system architecture, repetitive DNA, and natural genetic engineering. Gene 345, 91–100. 10.1016/j.gene.2004.11.020 15716117

[B153] Shinozaki K. Yamaguchi-Shinozaki K. Seki M. (2003). Regulatory network of gene expression in the drought and cold stress responses. Curr. Opin. Plant Biol. 6, 410–417. 10.1016/S1369-5266(03)00092-X 12972040

[B154] Sinzelle L. Izsvák Z. Ivics Z. (2009). Molecular domestication of transposable elements: from detrimental parasites to useful host genes. Cell. Mol. Life Sci. 66, 1073–1093. 10.1007/s00018-009-8376-3 19132291 PMC11131479

[B155] Slotkin R. Martienssen R. (2007). Transposable elements and the epigenetic regulation of the genome. Nat. Rev. Genet. 8, 272–285. 10.1038/nrg2072 17363976

[B156] Smith A. Hansey C. Kaeppler S. (2012). TCUP: a Novel hAT Transposon Active in Maize Tissue Culture. *Front*. Plant Sci. 3:6 10.3389/fpls.2012.00006 PMC3355664 22639634

[B157] Song W. Pi L. Bureau T. Ronald P. (1998). Identification and characterization of 14 transposon-like elements in the noncoding regions of members of the Xa21 family of disease resistance genes in rice. Mol. Gen. Genet. 258, 449–456. 10.1007/s004380050755 9669326

[B158] Song W. Pi L. Y. Wang G. L. Gardner J. Holsten T. Ronald P. C. (1997). Evolution of the rice Xa21 disease resistance gene family. Plant Cell 9, 1279–1287. 10.1105/tpc.9.8.1279 9286106 PMC156997

[B159] Strange A. Li P. Lister C. Anderson J. Warthmann N. Shindo C.. (2011). Major-effect alleles at relatively few loci underlie distinct vernalization and flowering variation in Arabidopsis accessions. PLoS ONE 6:e19949. 10.1371/journal.pone.0019949 21625501 PMC3098857

[B160] Studer A. Zhao Q. Ross-Ibarra J. Doebley J. (2011). Identification of a functional transposon insertion in the maize domestication gene tb1. Nat. Genet. 43, 1160–1163. 10.1038/ng.942 21946354 PMC3686474

[B161] Syed N. H. Sureshsundar S. Wilkinson M. J. Bhau B. S. Cavalcanti J. J. V. Flavell A. J. (2005). Ty1-copia retrotransposon-based SSAP marker development in cashew (*Anacardium occidentale* L.). Theor. Appl. Genet. 110, 1195–1202. 10.1007/s00122-005-1948-1 15761718

[B162] Tanaka A. (1999). Mutation induction by ion beams in *Arabidopsis*. Gamma Field Symp. 38, 19–27.

[B163] Tapia G. (2005). Involvement of ethylene in stress-induced expression of the TLC1.1 retrotransposon from *Lycopersicon chilense* dun. Plant Phys. 138, 2075–2086. 10.1104/pp.105.059766 16040666 PMC1183396

[B164] Tittel-Elmer M. Bucher E. Broger L. Mathieu O. Paszkowski J. Vaillant I.. (2010). Stress-induced activation of heterochromatic transcription. PLoS Genet. 6:e1001175. 10.1371/journal.pgen.1001175 21060865 PMC2965753

[B165] Tomato Genome Consortium (2012). The tomato genome sequence provides insights into fleshy fruit evolution. Nature 485, 635–641. 10.1038/nature11119 22660326 PMC3378239

[B166] Tsuchiya T. Eulgem T. (2013). An alternative polyadenylation mechanism co-opted to the Arabidopsis RPP7 gene through intronic retrotransposon domestication. Proc. Natl. Acad. Sci. U.S.A. 110, E3535–E3543. 10.1073/pnas.1312545110 23940361 PMC3773791

[B167] Turcich M. P. Bokhari-Riza A. Hamilton D. A. He C. Messier W. Stewart C. B. (1996). PREM-2, a copia-type retroelement in maize is expressed preferentially in early microspores. Sex. Plant Reprod. 9, 65–74. 10.1007/BF02153053

[B168] Uchiyama T. Saito Y. Kuwabara H. Fujino K. Kishima Y. Martin C.. (2008). Multiple regulatory mechanisms influence the activity of the transposon, Tam3, of *Antirrhinum*. New Phytol. 179, 343–355. 10.1111/j.1469-8137.2008.02477.x 19086175

[B169] Ungerer M. Strakosh S. Zhen Y. (2006). Genome expansion in three hybrid sunflower species is associated with retrotransposon proliferation. Curr. Biol. 16, R872–R873. 10.1016/j.cub.2006.09.020 17055967

[B170] van der Knaap E. A. Jackson S. A. Tanksley S. D. (2004). High-resolution fine mapping and fluorescence in situ hybridization analysis of sun, a locus controlling tomato fruit shape, reveals a region of the tomato genome prone to dna rearrangements. Genetics 168, 2127–2140. 10.1534/genetics.104.031013 15611181 PMC1448703

[B171] Vicient C. (2010). Transcriptional activity of transposable elements in maize. BMC Genomics 11:601. 10.1186/1471-2164-11-601 20973992 PMC3091746

[B172] Vicient C. Schulman A. (2002). Copia-like retrotransposons in the rice genome: few and assorted. Genome Lett. 1, 35–47. 10.1166/gl.2002.002

[B173] Vicient C. Suoniemi A. Anamthawat-Jónsson K. Tanskanen J. Beharav A. Nevo E.. (1999). Retrotransposon BARE-1 and its role in genome evolution in the genus *Hordeum*. Plant Cell 11, 1769–1784. 10.1105/tpc.11.9.1769 10488242 PMC144304

[B174] Volff J. (2006). Turning junk into gold: domestication of transposable elements and the creation of new genes in eukaryotes. Bioessays 28, 913–922. 10.1002/bies.20452 16937363

[B175] Vollbrecht E. Duvick J. Schares J. P. Ahern K. R. Deewatthanawong P. Xu L.. (2010). Genome-wide distribution of transposed dissociation elements in maize. Plant Cell 22, 1667–1685. 10.1105/tpc.109.073452 20581308 PMC2910982

[B176] Voronova A. Belevich V. Jansons A. Rungis D. (2014). Stress-induced transcriptional activation of retrotransposon-like sequences in the Scots pine (*Pinus sylvestris* L.) genome. Tree Genet. Genomes 10, 937–951. 10.1007/s11295-014-0733-1

[B177] Walbot V. (1999). UV-B damage amplified by transposons in maize. Nature 397, 398–399. 10.1038/17043 9989403

[B178] Walter J. Jentsch A. Beierkuhnlein C. Kreyling J. (2013). Ecological stress memory and cross stress tolerance in plants in the face of climate extremes. Environ. Exp. Bot. 94, 3–8. 10.1016/j.envexpbot.2012.02.009

[B179] Wang H. Chai Y. Chu X. Zhao Y. Wu Y. Zhao J.. (2009). Molecular characterization of a rice mutator-phenotype derived from an incompatible cross-pollination reveals transgenerational mobilization of multiple transposable elements and extensive epigenetic instability. BMC Plant Biol. 9:63. 10.1186/1471-2229-9-63 19476655 PMC2696445

[B180] White S. E. Habera L. F. Wessler S. R. (1994). Retrotransposons in the flanking regions of normal plant genes: a role for copia-like elements in the evolution of gene structure and expression. Proc. Natl. Acad. Sci. 91, 11792–11796. 10.1073/pnas.91.25.11792 7991537 PMC45321

[B181] Wicker T. Sabot F. Hua-Van A. Bennetzen J. L. Capy P. Chalhoub B.. (2007). A unified classification system for eukaryotic transposable elements. Nat. Rev. Genet. 8, 973–982. 10.1038/nrg2165 17984973

[B182] Witte C. Le Q. Bureau T. Kumar A. (2001). Terminal-repeat retrotransposons in miniature (TRIM) are involved in restructuring plant genomes. Proc. Natl. Acad. Sci. U.S.A. 98, 13778–13783. 10.1073/pnas.241341898 11717436 PMC61118

[B183] Woodrow P. Pontecorvo G. Fantaccione S. Fuggi A. Kafantaris I. Parisi D.. (2010). Polymorphism of a new Ty1-copia retrotransposon in durum wheat under salt and light stresses. Theor. Appl. Genet. 121, 311–322. 10.1007/s00122-010-1311-z 20237753

[B184] Xiong W. He L. Lai J. Dooner H. K. Du C. (2014). HelitronScanner uncovers a large overlooked cache of Helitron transposons in many plant genomes. Proc. Natl. Acad. Sci. U.S.A. 111, 10263–10268. 10.1073/pnas.1410068111 24982153 PMC4104883

[B185] Yaakov B. Ben-David S. Kashkush K. (2012). Genome-wide analysis of stowaway-like MITEs in wheat reveals high sequence conservation, gene association, and genomic diversification. Plant Physiol. 161, 486–496. 10.1104/pp.112.204404 23104862 PMC3532278

[B186] Yan Y. Zhang Y. Yang K. Sun Z. Fu Y. Chen X.. (2010). Small RNAs from MITE-derived stem-loop precursors regulate abscisic acid signaling and abiotic stress responses in rice. Plant J. 65, 820–828. 10.1111/j.1365-313X.2010.04467.x 21251104

[B187] Yao J. Dong Y. Morris B. A. (2001). Parthenocarpic apple fruit production conferred by transposon insertion mutations in a MADS-box transcription factor. Proc. Natl Acad. Sci. U.S.A. 98, 1306–1311 10.1073/pnas.98.3.1306 11158635 PMC14750

[B188] Yoshioka Y. Matsumoto S. Kojima S. Ohshima K. Okada N. Machida Y. (1993). Molecular characterization of a short interspersed repetitive element from tobacco that exhibits sequence homology to specific tRNAs. Proc. Natl. Acad. Sci. U.S.A. 90, 6562–6566. 10.1073/pnas.90.14.6562 8341669 PMC46972

[B189] Yu A. Lepère G. Jay F. Wang J. Bapaume L. Wang Y.. (2013). Dynamics and biological relevance of DNA demethylation in Arabidopsis antibacterial defense. Proc. Natl. Acad. Sci. U.S.A. 110, 2389–2394. 10.1073/pnas.1211757110 23335630 PMC3568381

[B190] Yu C. Zhang J. Peterson T. (2011). Genome rearrangements in maize induced by alternative transposition of reversed Ac/Ds termini. Genetics 188, 59–67. 10.1534/genetics.111.126847 21339479 PMC3120142

[B191] Zamboni A. Minoia L. Ferrarini A. Tornielli G. B. Zago E. Delledonne M.. (2008). Molecular analysis of post-harvest withering in grape by AFLP transcriptional profiling. J. Exp. Bot. 59, 4145–4159. 10.1093/jxb/ern256 19010774 PMC2639028

[B192] Zeller G. Henz S. R. Widmer C. K. Sachsenberg T. Rätsch G. Weigel D.. (2009). Stress-induced changes in the *Arabidopsis thaliana* transcriptome analyzed using whole-genome tiling arrays. Plant J. 58, 1068–1082. 10.1111/j.1365-313X.2009.03835.x 19222804

[B193] Zhang J. J. Zhou Z. S. Song J. B. Liu Z. P. Yang H. (2012). Molecular dissection of atrazine-responsive transcriptome and gene networks in rice by high-throughput sequencing. J. Hazard. Mater. 219–220, 57–68. 10.1016/j.jhazmat.2012.03.041 22503142

[B194] Zhang Q. Arbuckle J. Wessler S. (2000). Recent, extensive, and preferential insertion of members of the miniature inverted-repeat transposable element family Heartbreaker into genic regions of maize. Proc. Natl. Acad. Sci. U.S.A. 97, 1160–1165. 10.1073/pnas.97.3.1160 10655501 PMC15555

[B195] Zhang X. Feschotte C. Zhang Q. Jiang N. Eggleston W. B. Wessler S. R. (2001). P instability factor: an active maize transposon system associated with the amplification of Tourist-like MITEs and a new superfamily of transposases. Proc. Natl. Acad. Sci. U.S.A. 98, 12572–12577. 10.1073/pnas.211442198 11675493 PMC60095

[B196] Zhang X. Meng L. Liu B. Hu Y. Cheng F. Liang J.. (2015). A transposon insertion in FLOWERING LOCUS, T. is associated with delayed flowering in *Brassica rapa*. Plant Sci. 241, 211–220. 10.1016/j.plantsci.2015.10.007 26706072

